# Dynamics of natural and pharmacologic control of an SIV variant with an envelope trafficking defect

**DOI:** 10.1084/jem.20251172

**Published:** 2025-12-05

**Authors:** Kyle Rhodehouse, Carolin Zitzmann, Meghana Ravi, Ciara Armstrong, Milica Moskovljevic, Hannah P. Moore, Courtney Schill, Emily J. Fray, Mithra R. Kumar, Toni Penney, Clara Krzykwa, Miranda R. Stauss, Roger W. Wiseman, David H. O’Connor, Christine M. Fennessey, Brandon F. Keele, Jeffrey D. Lifson, Ruy M. Ribeiro, Alan S. Perelson, James A. Hoxie, Nicholas J. Maness, Janet D. Siliciano, Robert F. Siliciano

**Affiliations:** 1 Department of Medicine, The Johns Hopkins University School of Medicine, Baltimore, MD, USA; 2 Los Alamos National Laboratory, Los Alamos, NM, USA; 3 Tulane National Biomedical Research Center, Covington, LA, USA; 4 Wisconsin National Primate Research Center, University of Wisconsin-Madison, Madison, WI, USA; 5 AIDS and Cancer Virus Program, Frederick National Laboratory for Cancer Research, Frederick, MD, USA; 6 Perelman School of Medicine, University of Pennsylvania, Philadelphia, PA, USA; 7 Department of Microbiology and Immunology, Tulane University School of Medicine, New Orleans, LA, USA; 8 Howard Hughes Medical Institute, Baltimore, MD, USA

## Abstract

Insights into HIV-1 pathogenesis have come from studies of viral dynamics. However, there is little information on viral dynamics in lentiviral infections in which viral replication is naturally controlled in a subset of infected individuals. We evaluated the decay of simian immunodeficiency virus (SIV) RNA and cell-associated SIV genomes in a nonhuman primate (NHP) model in which replication of an engineered SIV variant is naturally controlled by cellular immune responses in most infected animals. This variant lacks a trafficking motif in the gp41 cytoplasmic tail. A trajectory of control was evident by 21 days after infection. In animals with natural control, we observed similar biphasic decay of intact proviruses in blood and lymph nodes, at rates close to those in animals that failed to control the virus and were put on antiretroviral therapy (ART). Both natural control and ART effectively blocked viral evolution, but not persistence. Thus, in this NHP model, natural control can be nearly as effective as ART in controlling viral replication.

## Teaser

Rhodehouse et al. characterize a nonhuman primate model of natural control of SIV infection in which the immune system blocks new infection events with an efficiency approaching that of antiretroviral therapy.

## Introduction

Important insights into the pathogenesis and treatment of HIV-1 infection have come from studies of viral decay dynamics (Ho et al., 1995; Wei et al., 1995; Perelson et al., 1996; Perelson et al., 1997). Following initiation of combination antiretroviral therapy (ART), new infection events are halted and the level of plasma virus decays rapidly to below the limit of detection (LOD) of clinical assays (Gulick et al., 1997; Hammer et al., 1997; Montaner et al., 1998; Hirsch et al., 1999). This decay is biexponential, representing the death, or transition to latency, of two distinct populations of virus-producing cells (Ho et al., 1995; Wei et al., 1995; Perelson et al., 1996; Perelson et al., 1997). Analysis of the decay of viral genomes in infected cells is complicated by the fact that most proviruses are defective due to deletions or APOBEC3-mediated hypermutation (Ho et al., 2013; Imamichi et al., 2016; Bruner et al., 2016; Bruner et al., 2019; Hiener et al., 2017; Lee et al., 2017; Bender et al., 2019; Peluso et al., 2020). Proviruses lacking these common defects can be measured with the intact proviral DNA assay (IPDA) (Bruner et al., 2019). This assay has been used to measure the on-ART decay of intact proviruses in CD4^+^ T cells in people with HIV-1 (PWH), as well as in simian immunodeficiency virus (SIV) and simian–human immunodeficiency virus (SHIV) infected nonhuman primates (NHPs) (White et al., 2022; Fray et al., 2023; Kumar et al., 2023). Biphasic decay is also observed, although the initial decay of cells with intact proviruses is slower than the rapid initial decay of plasma virus (White et al., 2022; Fray et al., 2023; Kumar et al., 2023). Following this initial decline of intact proviruses, there is a slower third phase of decay that does not become apparent until 2 years following ART initiation in NHPs (Fray et al., 2023). The stable latent reservoir decays very slowly, with a half-life of 3.7 years (Finzi et al., 1999; Siliciano et al., 2003; Strain et al., 2003; Crooks et al., 2015). After several years, decay further slows or ceases, and the reservoir may begin to slowly increase in size (Gandhi et al., 2023; McMyn et al., 2023), perhaps due to antigen-driven proliferation of infected cells (Mendoza et al., 2020; Simonetti et al., 2020; Moskovljevic et al., 2024).

Approximately 0.3% of PWH control viral replication through robust cellular immune responses. Termed elite controllers (ECs), these individuals maintain undetectable levels of plasma virus in the absence of ART (Deeks and Walker, 2007; Okulicz and Lambotte, 2011; Olson et al., 2014; Gonzalo-Gil et al., 2017; Yang et al., 2017; Capa et al., 2022; N’takpé et al., 2022). Like ART-treated PWH (Chun et al., 1995; Chun et al., 1997a; Chun et al., 1997b; Finzi et al., 1997; Wong et al., 1997), ECs exhibit persisting reservoirs of HIV-1 proviruses, although at levels approximately 1-log lower (Jiang et al., 2020; Kwaa et al., 2020). Recent studies have examined immune escape mutations and proviral integration sites in ECs (Jiang et al., 2020; Lian et al., 2021). While the dynamics of infected cell decay and persistence following ART initiation are well studied (Finzi et al., 1999; Siliciano et al., 2003; Strain et al., 2003; Crooks et al., 2015; Gandhi et al., 2023; McMyn et al., 2023), little is known about these parameters in the setting of immunologic control. Due to the rarity of ECs and the logistical challenges of studying acute infection, little is known about the dynamics of plasma virus, infected cells, and reservoir formation during the period when immunologic control is established. Limited data collected during acute and very early chronic HIV-1 infection of individuals who became ECs have indicated that plasma virus can become undetectable as early as 4–6 mo after infection (Goujard et al., 2009; Okulicz et al., 2009; Madec et al., 2013; Chen et al., 2014; Walker-Sperling et al., 2017; Moosa et al., 2018; Morley et al., 2019).

Viral control in this setting is, at least in part, mediated by critically timed, polyfunctional, highly effective cellular immune responses (Collins et al., 2020; Kwaa and Blankson, 2024). Although there are well-described models of immunologic control in macaques with particular MHC alleles (Evans et al., 1999; O’Connor et al., 2003; Loffredo et al., 2006; Loffredo et al., 2007; Yant et al., 2006; Mee et al., 2009; Aarnink et al., 2011; Budde et al., 2012; Mudd et al., 2012; Passaes et al., 2020), not all animals with these alleles control SIVs to below the LOD (Yant et al., 2006; Wojcechowskyj et al., 2007; Mudd et al., 2012), and studies to date have not been able to evaluate the dynamics of viral reservoir formation and maintenance as immunologic control is established.

To understand dynamics of viral reservoir formation and maintenance in the setting of natural control of viral replication, we used a NHP model in which pigtail macaques (*Macaca nemestrina*) (PTMs) are infected with an engineered derivative of the pathogenic SIVmac239 clone. The SIVmac239ΔGY virus (hereafter, ΔGY) lacks the glycine and tyrosine residues of a conserved GYxxΦ trafficking motif in the membrane-proximal region of the Env gp41 cytoplasmic tail (x = any a.a.; Φ = a.a. with a bulky hydrophobic side chain, i.e., ^720^GYRPV^724^ for SIVmac239, ^711^GYSPL^715^ for HIV-1 HXB2). This motif mediates clathrin-dependent endocytosis of Env from the plasma membrane (Rowell et al., 1995; Egan et al., 1996; Sauter et al., 1996; Ohno et al., 1997; Boge et al., 1998; Berlioz-Torrent et al., 1999) and likely contributes to virological synapse formation (Llewellyn et al., 2010; Wang et al., 2019). *In vitro*, this motif modulates Env surface expression on T cell lines (LaBranche et al., 1995; Sauter et al., 1996; Deschambeault et al., 1999) and directs Env basolateral sorting in polarized epithelial cells (Owens et al., 1991; Ball et al., 1997; Lodge et al., 1997). *In vivo*, loss of this motif profoundly alters the pathogenesis of SIVmac239 by sparing CD4^+^ T cells in gut lamina propria and failing to infect macrophages and the central nervous system (CNS) (Breed et al., 2013a; Breed et al., 2013b; Breed et al., 2015). During infection of PTMs, ΔGY replicates acutely to levels that are comparable to SIVmac239, but with the onset of host immune responses is controlled to low or undetectable levels in plasma, with animals remaining clinically well for months to years (Breed et al., 2013a; Breed et al., 2013b; Breed et al., 2015). ΔGY-control is associated with strong, polyfunctional antiviral CD4^+^ and CD8^+^ T cell responses, in the absence of neutralizing antibodies (Breed et al., 2015). A consistent finding in previous studies of ΔGY infected animals is that depletion of CD8α^+^ cells leads to transient spikes in viremia, reflecting the presence of persistent ΔGY reservoirs (Breed et al., 2015).

Here, we evaluated dynamics of viral reservoir formation and maintenance during natural control of ΔGY in PTMs. Although this model, involving an engineered virus, likely differs in important ways from elite control of HIV-1 in PWH, it does provide insights into viral dynamics. Interestingly, a subset of infected PTMs that failed to control ΔGY, and required ART for viral suppression, provided the opportunity to compare reservoir dynamics during natural versus pharmacologic control. We show that, in both groups, intact proviruses decayed with biexponential kinetics, similar to those reported during ART-treated SIVmac239 infection. These kinetics were identical in blood and lymph nodes (LNs). In both groups, little-to-no viral evolution was observed in reservoirs once immunologic or pharmacologic control was established. Our findings suggest that in this NHP model, natural control of viral replication can be as effective as ART in impacting the dynamics of reservoir formation and persistence.

## Results

### Cohort characteristics

**Figure 1.**
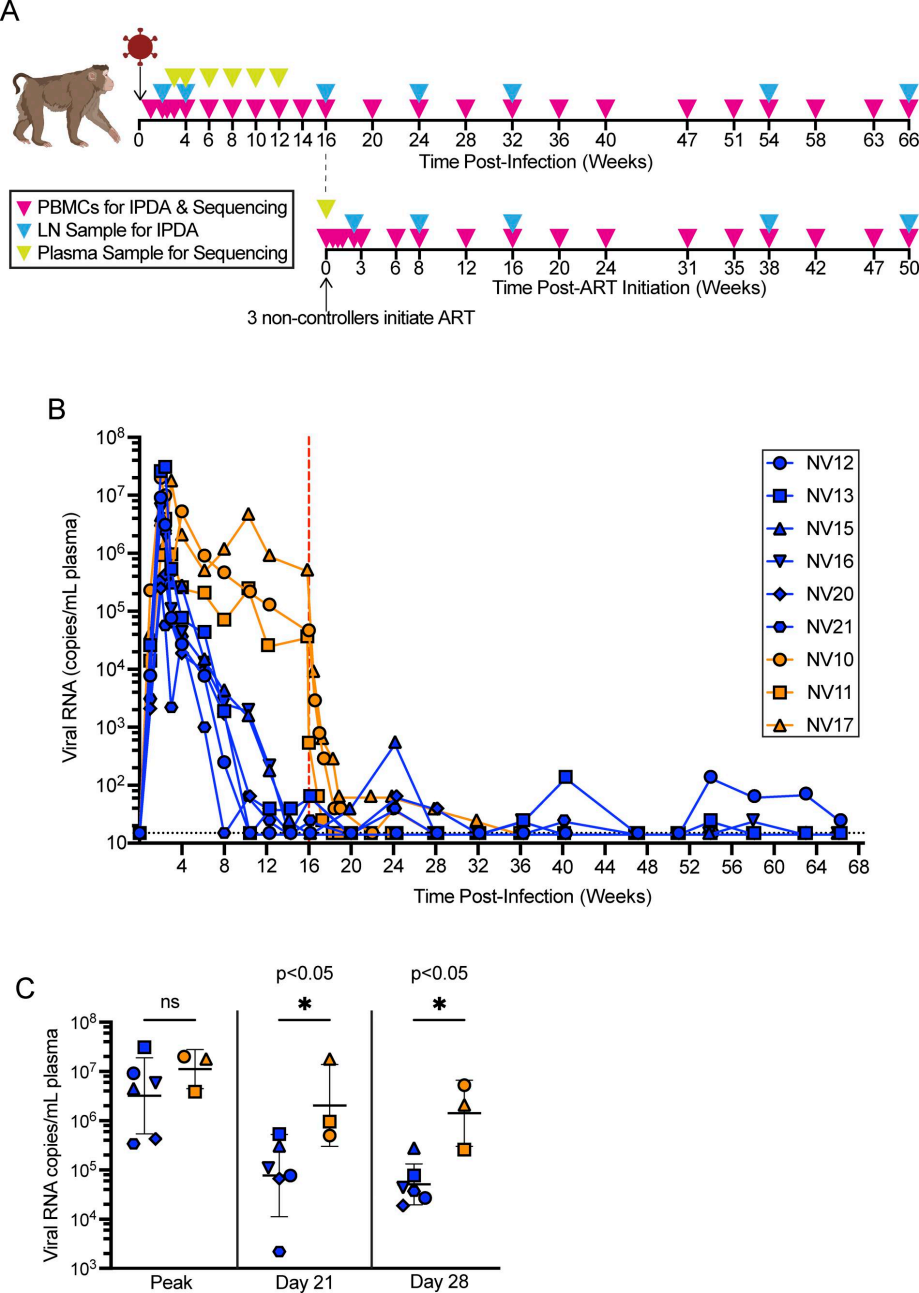
**Study design and plasma SIV RNA levels. (A)** Timeline of infection, ART initiation for ΔGY-non-controllers, and sampling of PBMCs (pink), peripheral LNs (blue), and plasma virus for sequencing (yellow). **(B)** Plasma SIV RNA levels through 66 wk after infection for controllers (blue) and non-controllers (orange). Each point is the mean of six technical replicates. The horizontal dotted line is the assay LOD (15 SIV RNA copies/ml). The vertical dotted line indicates ΔGY-non-controller ART initiation at 16 wk after infection. **(C)** Plasma SIV RNA measurements at peak viremia, day 21 after infection, and day 28 after infection. Shapes and colors are consistent with the key in B. Bars represent the geometric mean and geometric standard deviation. P values were determined using the Mann–Whitney nonparametric test.

We studied 9 PTMs (Table 1) infected intravenously with 2000 TCID_50_ barcoded ΔGY virus. Animals were inoculated with a high dose relative to previous studies (Breed et al., 2015) to ensure introduction of a diversity of barcoded viral variants. The genetic barcode allows for the identification and tracking of individual viral lineages over time for an otherwise clonotypic virus (Fennessey et al., 2017). We collected peripheral blood mononuclear cells (PBMCs), lymphocytes from peripheral LNs, and blood plasma throughout 66 wk of infection (Fig. 1 A).

**Table 1.** Cohort characteristics

Animal ID	Phenotype	Sex	Age at infection (years)	MHC-A Haplotype 1	MHC-A Haplotype 2	MHC-B Haplotype 1	MHC-B Haplotype 2
NV10	Non-controller	F	3.5	A019g2	A032	B016b	B004b
NV11	Non-controller	F	3.1	A019g2	A032	B016b	B016b
NV17	Non-controller	F	4.1	A019g2	A052	B016b	B099
NV12	Controller	F	3.5	A009	A084	B024b	B028
NV13	Controller	M	4.3	A010	A084	B017e	B122
NV15	Controller	M	3.8	A114	A084	B120c	B111b
NV16	Controller	F	4.8	A019_03	A084	B047a	B069b
NV20	Controller	M	3.6	A019g1	A082	B047a	B043
NV21	Controller	M	3.0	A052	A084	B099	B015b

M, male; F, female.

### Emergence of controllers and non-controllers

Peak viral loads (geometric mean: 4.9 × 10^6^ SIV RNA copies/ml) (Fig. 1 B) were comparable to those of animals infected with SIVmac239 (Breed et al., 2015). Six animals exhibited a decline in viremia to levels typically seen during natural control of ΔGY (Breed et al., 2015). However, plasma virus levels declined more slowly in three animals (NV10, NV11, and NV17). Generally, control of viremia to <100 copies of SIV RNA copies/ml occurs within the first 15 wk of ΔGY infection of PTMs. NV10, NV11, and NV17 all had plasma SIV RNA levels above 10,000 copies/ml at 16 wk after infection, and were considered to be non-controllers. Further evidence that these animals were unlikely to eventually develop control of viral replication comes from the finding that upon treatment interruption at 95 wk after infection, all three animals experienced viral rebound and were unable to control viral replication, maintaining post-ART set points >1,000 RNA copies/ml (not shown). Given that some ΔGY infected PTMs and rhesus macaques (RMs) develop persistent viremia and fail to control this virus (Breed et al., 2013a; Breed et al., 2015; Lawrence et al., 2022), these three animals initiated ART at 16 wk after infection (see Materials and methods). Lack of ΔGY-control in this subgroup gave us the opportunity to compare the dynamics of viral reservoir formation and maintenance during elite immunologic control (in NV12, NV13, NV15, NV16, NV20, and NV21) and ART-mediated, pharmacologic control (in NV10, NV11, and NV17).

For ΔGY-controllers, the median time difference from peak viral load to first undetectable measurement (i.e., <15 copies/ml) was 71 days (range: 42–99 days). Most experienced transient blips (median: 65 copies/ml, range: 25–540 copies/ml), before achieving undetectable measurements. ΔGY-non-controllers exhibited a rapid decline in viral load following ART initiation and reached undetectable levels after a median of 42 days (range: 17–141 days) following ART initiation. ΔGY-controllers had a half-log lower peak viral load than the ΔGY-non-controllers (Fig. 1 C), but this difference was not statistically significant (controllers: geometric mean 3.2 × 10^6^ RNA copies/ml, non-controllers: geometric mean 1.1 × 10^7^ RNA copies/ml, P = 0.55). Peak viremia was reached on day 14 or 17 by all animals, except NV17 (on day 21). Importantly, plasma virus levels in the non-controllers diverged significantly from levels in the controllers by day 21 after infection (ΔGY-controllers: geometric mean 7.7 × 10^4^; ΔGY-non-controllers: geometric mean 2.1 × 10^6^, P = 0.048) (Fig. 1 C). Because NV17’s viral load also peaked on day 21, we repeated this comparison using data from day 28 (7–14 days post-peak viral load, controllers: geometric mean 5.1 × 10^4^; non-controllers: geometric mean 1.4 × 10^6^, P = 0.048). This indicates that immunologic control is established early, within the first 3–4 wk of infection.

### Decay of viremia

Previous studies demonstrated that following ART initiation, plasma HIV-1 RNA levels decay in a biexponential manner, representing the death or transition to latency of two distinct populations of cells that produce the majority of the plasma virus (Perelson et al., 1997). For HIV-1, the first-phase decay has a half-life of <1 day (Ho et al., 1995; Wei et al., 1995; Perelson et al., 1996; Perelson et al., 1997) and is evident for ∼2 wk, during which time plasma HIV-1 RNA levels decay to ∼1% of pre-ART values (Perelson et al., 1996; Perelson et al., 1997; White et al., 2022). The second phase, representing the decay of the cells producing most of the remaining 1% of plasma virus, is slower and more variable (on a span of days to weeks) (Perelson et al., 1997; White et al., 2022). Crucially, the reason for the biphasic mode of decay has never been established; the identities of the two populations of cells, as well as their locations in the body, remain unclear. Of note, no studies to date have captured the early decay kinetics of plasma virus in ECs. Thus, our cohort provided the opportunity to directly compare the kinetics of plasma virus decline in PTMs infected with the same virus, but experiencing immune-mediated elite control or ART-mediated pharmacologic control.

**Figure 2.**
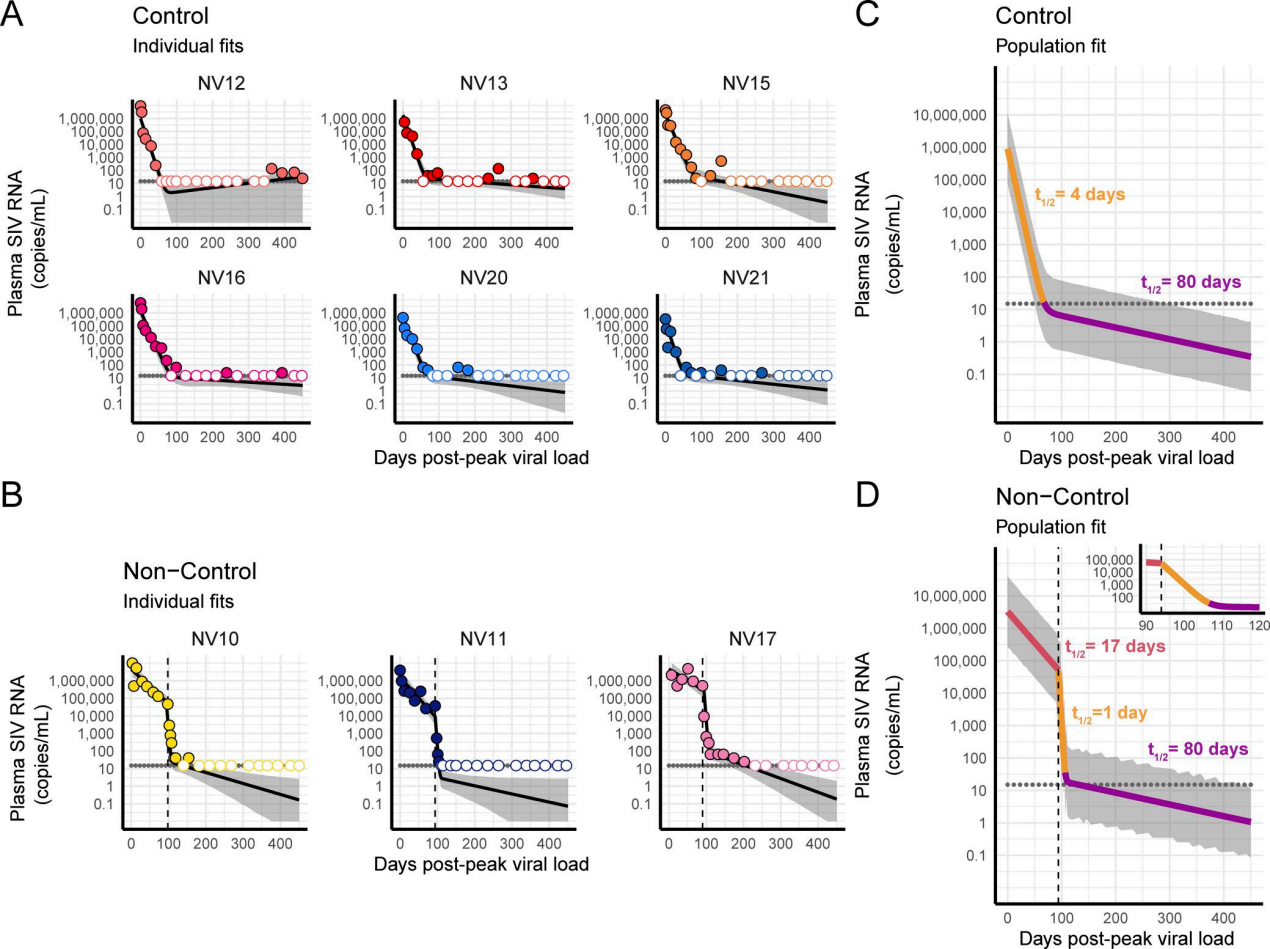
**Decay of plasma virus. (A–D)** Individual best fits for each ΔGY-controller (A) and ΔGY-non-controller (B) and population best fits for ΔGY-controllers (C) and ΔGY-non-controllers (D). Each decay phase is indicated by a color, with corresponding half-life indicated in respectively colored text. Each point is the mean of six technical replicates. Open circles represent values below the LOD of the assay (15 SIV RNA copies/ml), which is indicated by a horizontal dotted line. Gray shading represents 95% confidence bands. A vertical dotted line represents the date of ART initiation. Insert graph in D is a zoomed-in version of the main graph.

We used nonlinear mixed-effects modeling to quantitatively assess differences in decay kinetics of SIV RNA in blood plasma between ΔGY-controllers and ΔGY-non-controllers. In this approach, we fit the data from the two groups of animals simultaneously and assessed the statistical differences in decay rates using a covariate. In both groups, the decay of virus from peak (for ΔGY-controllers) or from day of ART initiation (for ΔGY-non-controllers) was best described by a biphasic, exponential decrease. The individual best fits for each animal are shown in Fig. 2 A (ΔGY-controllers) and Fig. 2 B (ΔGY-non-controllers), and the population best fits are shown in Fig. 2, C and D. Data associated with all the following calculations are available in Tables S1 and S2.

In ΔGY-controllers, plasma virus levels decayed from peak, presumably due to the onset of host immune responses, with a first-phase half-life of 4 days (95% confidence interval [CI]: 3.3–5 days), followed by a second decay phase with a half-life of 80 days (95% CI: 40.8–762 days) (Fig. 2 C). In ΔGY-non-controllers, prior to ART, plasma virus decayed with a half-life of 17 days (95% CI: 9.2–165 days), and upon ART initiation, biphasic decay was observed with a first-phase half-life of 1 day (95% CI: 0.8–1.6 days), followed by a second phase, which, perhaps due to lack of data above the LOD of the assay, was not statistically different from the ΔGY-controllers, computed with a half-life of 80 days (95% CI: 40.8–762 days) (Fig. 2 D).

The significant difference in the first-phase decay of plasma virus in ΔGY-controllers (*t*_*1/2*_ = 4 days) and initial pre-ART decay of plasma virus in ΔGY-non-controllers (*t*_*1/2*_ = 17 days, P = 1.95 × 10^−7^) suggests that the factors responsible for elite control are initiated early in infection.

Another important observation is that the first phase of decay of plasma virus after ART in ΔGY-non-controllers was significantly faster (*t*_*1/2*_ = 1 day) than the post-peak first-phase decay of plasma virus in ΔGY-controllers (*t*_*1/2*_ = 4 days, P = 0.024), which likely reflects the complete cessation of infection events following ART initiation (Joos et al., 2008; Evering et al., 2012; Josefsson et al., 2013; Oue et al., 2013; Kearney et al., 2014; Kearney et al., 2015; Kearney et al., 2017; Brodin et al., 2016; Rosenbloom et al., 2017; Van Zyl et al., 2017; Mok et al., 2018; Bozzi et al., 2019; Cadena et al., 2021; Fray et al., 2023; Immonen et al., 2024; Lee et al., 2024), while some replication, at least initially, continues during immunologic control (Bailey et al., 2006a; Bailey et al., 2006b; Miura et al., 2009; Mens et al., 2010; O’Connell et al., 2010; Salgado et al., 2010; Fukazawa et al., 2015; Boritz et al., 2016; Casado et al., 2020). Nevertheless, that post-peak first-phase decay of plasma virus in ΔGY-controllers and initial on-ART decay of plasma virus in ΔGY-non-controllers both occur in the span of days indicates that, in this model, the immune system blocks new infection events with an efficiency approaching that of ART.

### Decay of intact proviruses in peripheral blood

In ART-treated PWH, the overwhelming majority of infected cells harbor a provirus that is defective due to large internal deletions and/or APOBEC3-mediated hypermutation (Ho et al., 2013; Bruner et al., 2016; Imamichi et al., 2016; Hiener et al., 2017; Lee et al., 2017). Intact and defective proviruses are subjected to different selective pressures, and so have different dynamics *in vivo*, with more rapid decay of intact proviruses and persistence of defective proviruses (Peluso et al., 2020; White et al., 2022). Thus, to understand the dynamics of intact and defective proviruses in ΔGY-controllers and ΔGY-non-controllers, we used the IPDA to selectively quantify CD4^+^ T cells harboring proviruses that lack common fatal defects (Bruner et al., 2019; Bender et al., 2019; Kumar et al., 2023). IPDA values are corrected for DNA shearing and the presence of 2–long terminal repeat (2LTR) circles.

**Figure 3.**
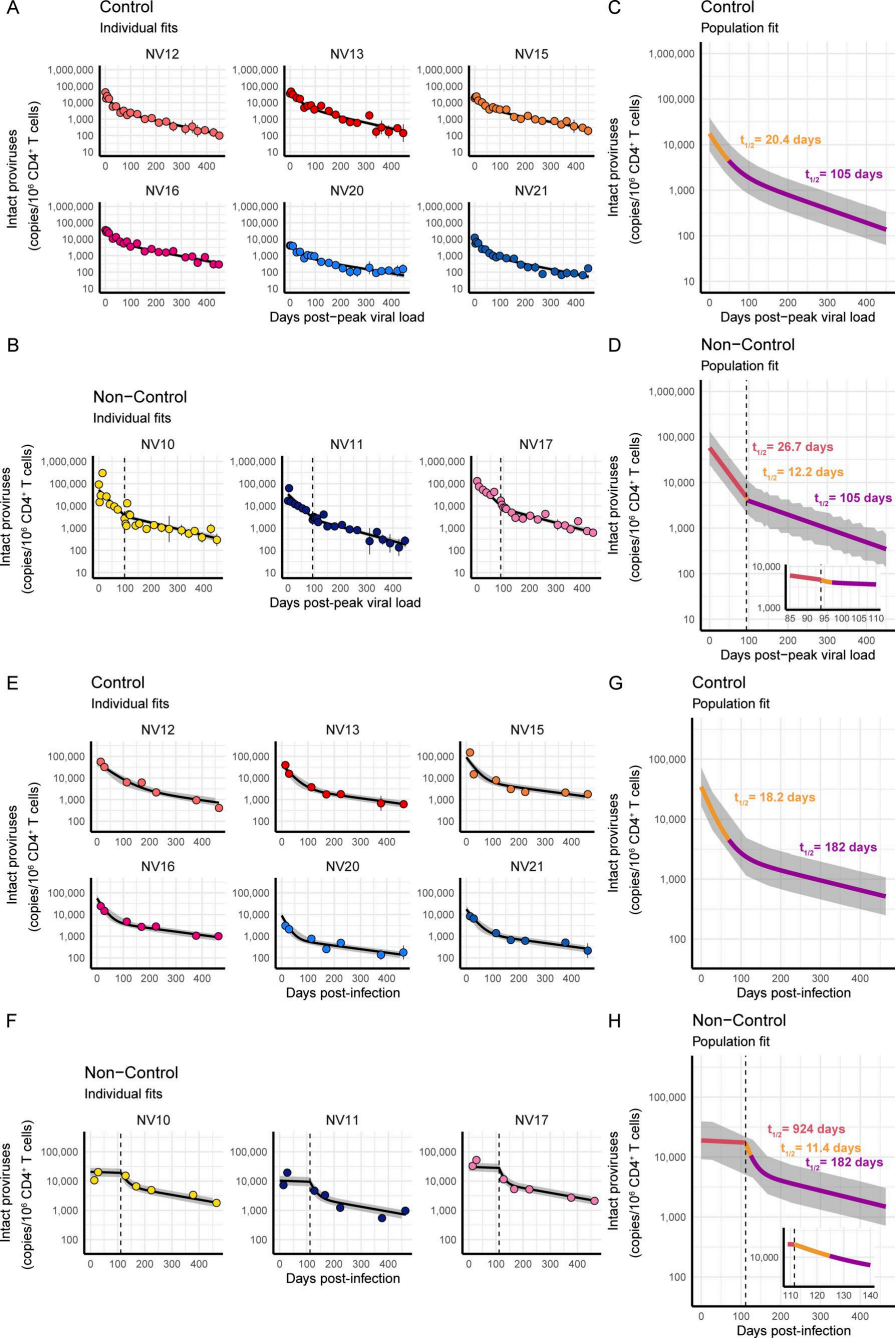
**Decay of intact proviruses in peripheral blood and LNs. (A–D)** Results from peripheral blood. Individual best fits for each ΔGY-controller (A) and ΔGY-non-controller (B) and population best fits for ΔGY-controllers (C) and ΔGY-non-controllers (D). **(E–H)** Results from LNs. Individual best fits for each ΔGY-controller (E) and ΔGY-non-controller (F) and population best fits for ΔGY-controllers (G) and ΔGY-non-controllers (H). Each point is the geometric mean of ≥5 technical replicates, corrected for DNA shearing and *env*+ 2LTR circles and normalized per 10^6^ CD4^+^ T cells. Bars are geometric standard deviation. Each decay phase is indicated by a color, with corresponding half-life indicated in respectively colored text. Gray shading represents 95% confidence bands. Vertical dotted lines represent ART initiation. Insert graphs in D and H are zoomed-in versions of the main graph.

The decay of intact proviruses, from the acute viral peak in ΔGY-controllers, or from the day of ART initiation in ΔGY-non-controllers, was best described by a biexponential decrease. This decay may represent the death, or exit from circulation, of cells containing intact provirus. The individual best fits of decay for each animal are shown in Fig. 3, A and B. In ΔGY-controllers, intact proviruses decayed with a first-phase half-life of 20.4 days (95% CI: 18.2–22.4 days) and a second-phase half-life of 105 days (95% CI: 92–124 days) (Fig. 3 C). In ΔGY-non-controllers, prior to ART, intact proviruses decayed with a half-life of 26.7 days (95% CI: 23.1–31.5 days). Thus, the initial decay of intact proviruses in ΔGY-non-controllers was significantly slower (*t*_*1/2*_ = 26.7 days) than the initial decay of intact proviruses in ΔGY-controllers (*t*_*1/2*_ = 20.4 days, P = 5.1 × 10^−4^).

Upon ART initiation in ΔGY-non-controllers, intact proviruses decayed with a first-phase half-life of 12.2 days (95% CI: 4.3–+15.1 days). The + represents a doubling time. Thus, the CI includes no decay. The wide CIs reflect the small number of non-controllers, biological variability in the frequency of infected cells in the circulation, and the short time interval over which this initial phase of on-ART decay was defined by the best-fit model. The second-phase half-life was 105 days (95% CI: 92–124 days), not statistically different from that of the ΔGY-controllers (Fig. 3 D).

As seen with the decay of plasma virus, the first-phase decay of intact proviruses following ART initiation in ΔGY-non-controllers (*t*_*1/2*_ = 12.2 days) is significantly faster than the first-phase decay of intact proviruses in ΔGY-controllers (*t*_*1/2*_ = 20.4 days, P = 0.024). This again likely reflects the lesser degree to which the immune system, at least initially, prevents the infection of new cells relative to complete pharmacologic inhibition. That these half-lives are within a range of 10–20 days demonstrates the potency of the ensuing immune response as the ΔGY-controllers transition to elite control.

Consistent with previous observations in ART-treated PWH (White et al., 2022), these first-phase decay rates, post-peak or post-ART initiation, are much slower (5.1× for controllers, 12.2× for non-controllers) than the first-phase decay of the plasma virus. This again suggests that the cells producing most of the plasma virus are not present in circulation, represent only a small fraction of the circulating cells harboring intact proviruses, or rapidly transition from highly productive infection to latency. To this last hypothesis, an experimental model recapitulating the establishment of HIV-1 latency demonstrated that latency results primarily from infection of cells in an effector-to-memory state transition. These cells concurrently downregulate the expression of NF-κB–dependent genes and are less permissive to viral transcription (Shan et al., 2017). Thus, most virus production *in vivo* is unlikely to originate from these transitioning cells.

### Decay of intact proviruses in peripheral LNs

CD4^+^ T cells are found throughout the body, with only ∼2% in the circulation (Trepel, 1974; Westermann and Pabst, 1992; Reinhardt et al., 2001; Di Mascio et al., 2009). The contributions of different anatomical sites, both lymphoid and nonlymphoid tissues, to the latent reservoir remain a key concern for HIV cure efforts (Wong and Yukl, 2016). The discrepancies in the half-lives of plasma virus and circulating cells containing intact provirus suggest that the cells producing most of the plasma virus are not in circulation (White et al., 2022). These cells may be localized to secondary lymphoid tissues, including the peripheral LNs. CD4^+^ T cells that enter the paracortex of a LN encounter antigen presented by dendritic cells, resulting in T cell activation (i.e., a highly permissive state for viral replication) (Moskovljevic et al., 2024) within an environment that encourages temporary retention in the LN (Von Andrian and Mempel, 2003; Schwab and Cyster, 2007; Mandl et al., 2012; Masopust and Schenkel, 2013; Griffith et al., 2014; Groom, 2015).

**Figure 4.**
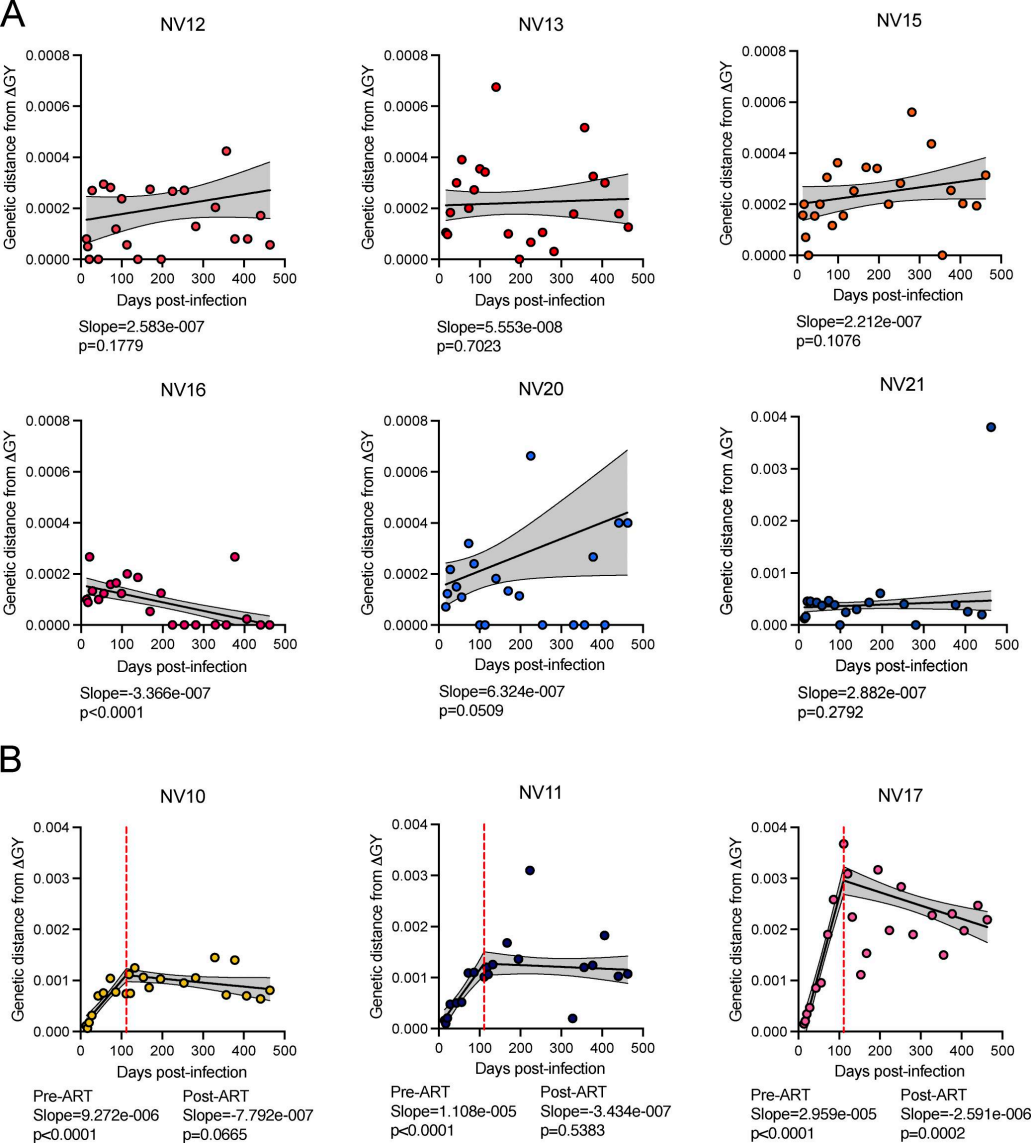
**Lack of ongoing evolution in ΔGY-controllers. (A)** Linear regression analyzed the pairwise distance (p-distance) as a function of time in controllers. Regressions were fit from peak viral load of each animal to week 66. **(B)** Segmental linear regression analyzed the p-distance as a function of time in non-controllers. Regressions were fit from day 14 to week 66, with transition point constrained to ART initiation, represented by a dotted red line. Defective proviruses were excluded. p-distance was calculated using the maximum composite likelihood model. p-distance of ∼0.0004 corresponds to a difference of 1 nucleotide. Each point is the mean p-distance for a time point (*n* = 1–57). An extra-sum-of-squares F test determined whether slope was significantly different from 0. Slopes and P-values are indicated under the respective graph. The gray-shaded area bounded by dotted lines is the 95% CI.

To test this hypothesis, we performed the IPDA on lymphocytes obtained by excisional biopsy of peripheral (axillary or inguinal) LNs throughout the 66 wk of the experiment (Fig. 1 A). The individual best fits of decay of intact proviruses for each animal are shown in Fig. 3 E and Fig. 4 F, and the best fits of decay for each animal group are shown in Fig. 3, G and H. Surprisingly, we observed that the decay of intact proviruses was similar in blood and LNs, for both ΔGY-controllers and ΔGY-non-controllers.

In ΔGY-controllers, from the acute viral peak, intact proviruses in LNs decayed with a first-phase half-life of 18.2 days (95% CI: 7.5–43.3 days) and a second-phase half-life of 182 days (95% CI: 136–257 days) (Fig. 3 G). In ΔGY-non-controllers, upon ART initiation, intact proviruses in LNs decayed with a first-phase half-life of 11.4 days (95% CI: 2.6–49.5 days) and a second-phase half-life of 182 days (CI: 136–257 days) (Fig. 3 H). The first phase of decay of intact proviruses following peak viremia in controllers or following ART initiation in non-controllers was nearly identical between blood and LNs (*t*_*1/2*_ = 20.4 days in blood and 18.2 days in LN for controllers, P = 0.39; 12.2 days in blood and 11.4 days in LN for non-controllers). Thus, first-phase decay of intact proviruses in the LN was also much slower than first-phase decay of the plasma viremia. This suggests that the cells producing most of the plasma virus do not reside in the peripheral LNs, or represent only a very small fraction of the LN cells carrying intact proviruses.

### Decay of 2LTR circles and hypermutated proviruses

2LTR circles are unintegrated viral genomes that form when end–end ligation, rather than integration, occurs following reverse transcription. While replication-defective, they are relatively stable (Sharkey et al., 2000; Sharkey et al., 2005; Butler et al., 2002; Pierson et al., 2002; Policicchio et al., 2018), thus complicating PCR-based measures of intact proviruses (Bender et al., 2019; White et al., 2022; Fray et al., 2023). The SIV IPDA includes measurements of 2LTR circles (Butler et al., 2002; Pierson et al., 2002; Bender et al., 2019; Policicchio et al., 2018) and allows for separate quantification of 2LTR circles that have an intact *env* region, and those with a deletion in *env*. In both groups of animals, we observed decay kinetics for 2LTR circles that were similar to those for intact proviruses (Tables S1 and S2), consistent with previous observations for SIV (Fray et al., 2023), SHIV (Kumar et al., 2023), and HIV-1 (White et al., 2022). *env+* circles decayed faster than *env*- circles. 2LTR circles can express viral transcripts and proteins (Wu and Marsh, 2003; Sloan et al., 2010; Bonczkowski et al., 2016; Policicchio et al., 2018), and thus, cells containing them may be targeted by the immune system.

In ΔGY-non-controllers, we observed a brief, but steep, drop in the quantity of 2LTR circles following ART initiation (Tables S1 and S2). Previous studies have not included extensive pre-ART sampling (Bender et al., 2019; White et al., 2022; Fray et al., 2023; Kumar et al., 2023), and thus, this decay pattern may have been missed. While inhibition of reverse transcription (through treatment with two RT inhibitors, tenofovir and emtricitabine, see Materials and methods) would preclude the formation of new 2LTR circles, it does not explain why the drop in quantity of 2LTR circles is so rapid, as treatment should not affect the stability of previously generated 2LTR circles. Treatment with an integrase inhibitor (dolutegravir) has been linked to a transient increase in the quantity of 2LTR circles (Buzón et al., 2010; Hatano et al., 2013b; Luo et al., 2013; Martinez-Picado et al., 2018). We also modeled the decay of 2LTR circles in the LNs (Tables S1 and S2). Because we did not have time points spaced as closely as in blood, we did not observe a steep drop in 2LTR circles upon ART initiation.

Unlike the HIV-1 IPDA, the SIV IPDA uses unlabeled competition probes at both amplicons to discriminate hypermutated sequences. As a result, the assay is not quantitative with regard to 3′ or 5′ defective proviruses (Bender et al., 2019). Thus, we could not determine the decay of these defective proviruses, which for HIV-1 was shown to be monophasic (White et al., 2022). Instead, we used the recently developed hypermutated proviral DNA assay (HPDA) to quantify one type of defective SIV proviruses—hypermutants (Fray et al., 2023). Contrary to the previous assessment of hypermutated provirus decay kinetics in SIVmac251-infected RMs (Fray et al., 2023), the decay in our cohort was best described by a biphasic, exponential decrease with very long second-phase half-lives (Tables S1 and S2). Hypermutation results in proviruses riddled with missense and nonsense mutations that are unable to be translated into full-length viral proteins (Bender et al., 2019), limiting surveillance by the immune system. The slow decay observed here, and in the initial study that described the HPDA (Fray et al., 2023), is reflective of a lack of immune clearance and cellular proliferation.

### Low viral diversity in the setting of immunologic control

To understand qualitative changes in virus populations in the ΔGY-model, we performed sequencing of plasma viruses and proviruses from blood across the 66 wk of the study. Using a novel near-full-length SIV genome sequencing assay Sequencing of Macaque-Integrated Lentiviruses (SMILe), based on MIP-Seq (Einkauf et al., 2019), we collected 151 near-full-length proviral sequences. Among these, 136 were intact, as expected given that only proviruses with successful amplification of all four subgenomic amplicons were sequenced. To more fully evaluate viral evolution in this model, we also obtained 3,142 *env* sequences from PBMCs and 409 *env* sequences from plasma.

Most ECs experience persistent low-level viremia (Bailey et al., 2006a; Dinoso et al., 2008; Hatano et al., 2009; Pereyra et al., 2009), like ART-treated non-controllers (Dornadula et al., 1999; Palmer et al., 2003; Maldarelli et al., 2007; Dinoso et al., 2008). Unlike residual viremia in ART-treated non-controllers, which reflects the stochastic reactivation of latently infected cells, the low-level viremia in ECs may also reflect a low level of ongoing viral replication, albeit at such low levels that viruses do not significantly reinfect and reseed the reservoir (Bailey et al., 2006a, 2006b). This is reflected by the limited proviral diversity seen in ECs, compared with the more extensive sequence diversity observed in non-controllers (Bailey et al., 2006a; Bailey et al., 2006b; Miura et al., 2009; Mens et al., 2010; O’Connell et al., 2010; Salgado et al., 2010; Fukazawa et al., 2015; Boritz et al., 2016; Casado et al., 2020).

We found that the ΔGY-model replicates this phenotype. After excluding sequences with hypermutation, nonsense mutations, and frame-shifting length polymorphisms, we analyzed 3,564 *env* sequences. To determine whether genetic distance from the inoculum increased over time, we performed linear regressions to estimate the slope of the line of average genetic distance as a function of time. Among the ΔGY-controllers, five animals had slopes not significantly different from zero, while NV16 had a negative slope (Fig. 4 A). For the ΔGY-non-controllers, we performed segmental linear regressions, with the inflection point at the time of ART initiation. As expected, all ΔGY-non-controllers had significant positive slopes before ART, indicating ongoing viral evolution resulting from ongoing replication. After ART initiation, the slopes for NV10 and NV11 were not significantly different from zero, indicating a lack of evolution on ART; NV17 had a significant negative slope (Fig. 4 B). The negative slopes for NV16 and NV17 reflect the decay of the cells containing more divergent variants (Brooks et al., 2020; Fray et al., 2023).

**Figure 5.**
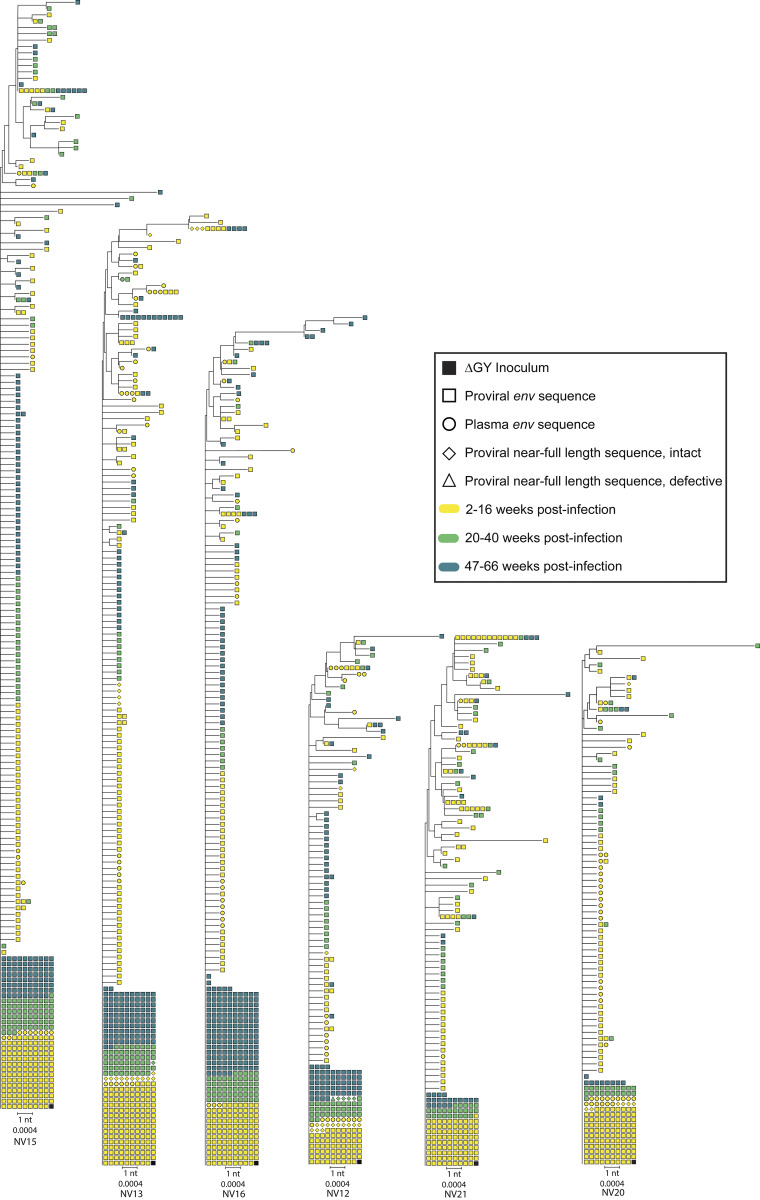
**Phylogenetic trees of *env* sequences from ΔGY-controllers.** Trees excluded sequences with hypermutation, nonsense mutations, and frame-shifting length polymorphisms in *env*. Colors delineate time point, and shapes represent sequence types. The scale bar represents 1 nucleotide (nt), corresponding to p-distance of 0.0004. Because of small, in-frame deletions, the number of nucleotides analyzed for each tree differs slightly.

**Figure 6.**
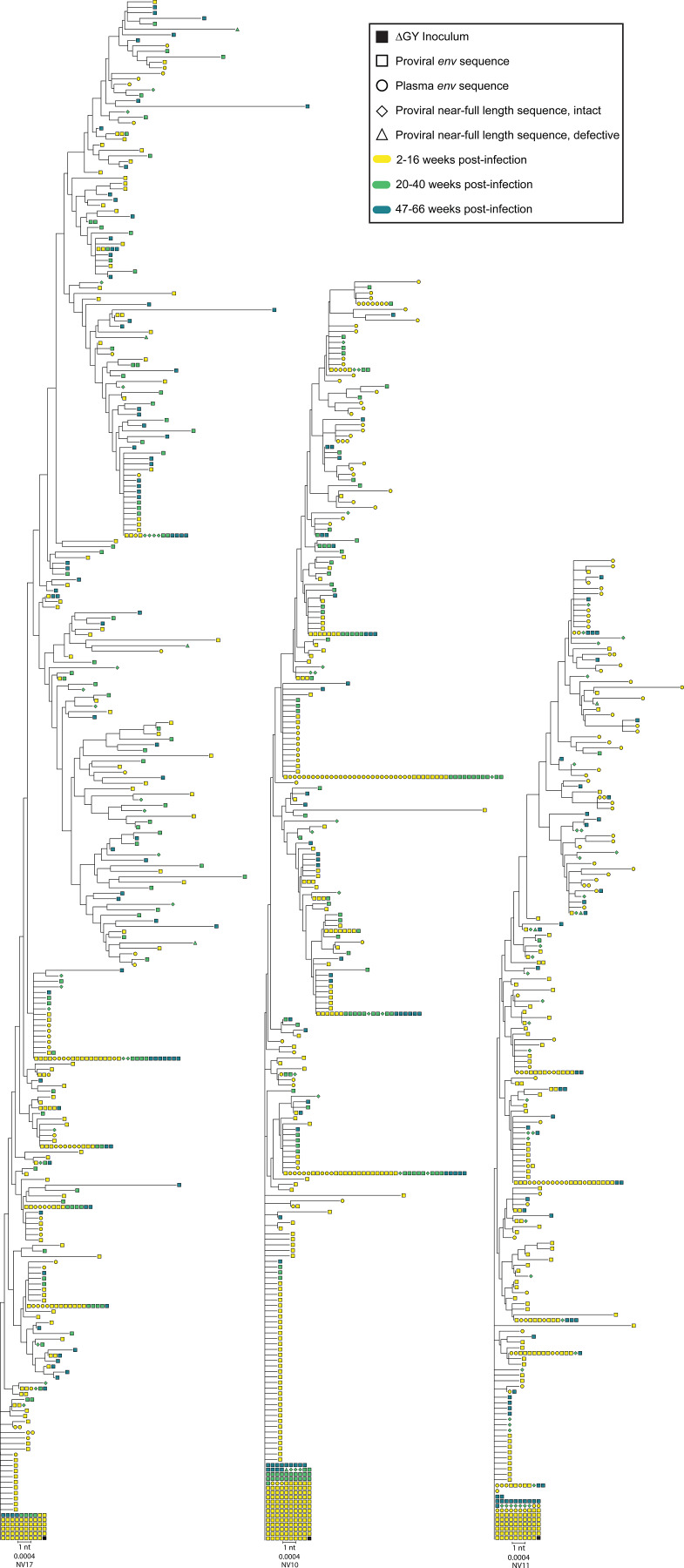
**Phylogenetic trees of *env* sequences from ΔGY-non-controllers.** Trees excluded sequences with hypermutation, nonsense mutations, and frame-shifting length permutations in *env*. Colors delineate time point, and shapes represent sequence types. Scale bar represents 1 nucleotide (nt), corresponding to p-distance of 0.0004. Because of small, in-frame deletions, the number of nucleotides analyzed for each tree differs slightly.

We constructed phylogenetic trees for each animal to analyze evolutionary relationships between sequences (Figs. 5 and 6). As in all previous studies, the ΔGY deletion was universally conserved (Breed et al., 2013a; Breed et al., 2013b; Breed et al., 2015). For the ΔGY-controllers, the majority (69%) of all intact cellular *env* sequences were identical to the inoculum, and this fraction was consistent at each time point. These proviruses must have been archived early in infection, as the low fidelity of reverse transcriptase assures mutation as a byproduct of replication (Coffin, 1995). Most sequences not identical to the inoculum were arranged into a comblike structure, characterized by a lack of branching and few shared mutations between sequences (Fig. 5). A minority of sequences were arranged into defined clades, but sequences collected earlier were typically not ancestral to sequences collected later. Sequences collected later were found throughout the branches, and were, at times, identical to sequences collected earlier (Figs. 5 and 6). In the controllers, we observed a maximum of only 12 nucleotide changes in any given sequence; only 6 or fewer nucleotide changes were observed in more than 3 individual sequences. These results, as well as the linear regression analysis (Fig. 4), collectively establish that in the ΔGY-model, control is sufficiently strong so as to halt most ongoing replication and limit accumulation of genetic diversity. However, we cannot exclude the possibility that more extensive sequencing would identify rare variants that have diverged further from the founder sequence. The phylogenetic patterns observed are consistent with those of NHPs and PWH initiating ART very soon following infection (Evering et al., 2012; Immonen et al., 2024). Additional evidence for a lack of ongoing replication is the conspicuous absence of an R751G substitution in most ΔGY-controller sequences, even among those with defined branching. This mutation, which corrects a suboptimal position in the lab-generated SIVmac239 clone, is a strong correlate of viral replication and typically becomes fixed within days of infection (Alexander et al., 2001; Fennessey et al., 2015). In contrast, the ΔGY-non-controllers displayed many fewer sequences identical to the inoculum (28% of all intact cellular *env* sequences) and displayed a pattern of branching and clustering consistent with an accumulation of proviral diversity in the 16 wk prior to ART initiation (Fig. 6).

Interestingly, because of the early control of viral replication, many of the independent *env* sequences from each animal were identical (Figs. 5 and 6). Clonality is difficult to assess without integration site data (Sherman et al., 2017; Patro et al., 2019; Wells et al., 2020), and we expect that many of the identical *env* sequences detected were derived from cells infected with a dominant early variant or founder sequence. Within the first few weeks of infection, it is unlikely that the proliferation of infected cells could generate infected cell clones large enough to dominate the proviral population. Indeed, prominent infected cell clones were not observed in previous studies of SIV-infected RMs that initiated ART after 1–2 years of infection (Bender et al., 2019; Fray et al., 2023).

### Cytoplasmic tail mutations

Previous instances of ΔGY-non-control were characterized by the evolution of, and selection for, novel trafficking signals in the cytoplasmic tail of gp41, which restored endocytic trafficking and/or basolateral sorting functions (Breed et al., 2013b; Lawrence et al., 2022). However, in the ΔGY-non-controllers in the current study, we did not observe mutations in any sequence that generated a new YxxΦ motif, nor did we observe mutations that generated a new dileucine motif ([D/E]xxxL[L/I] or DxxLL), which also mediates endocytic trafficking of cellular proteins (Bonifacino and Traub, 2003). Another important gp41-based trafficking signal is the C-terminal noncanonical dileucine motif (^878^LL^879^ SIVmac239 numbering, ^855^LL^856^ HXB2 numbering), which, like the membrane-proximal YxxΦ motif (Rowell et al., 1995; Egan et al., 1996; Sauter et al., 1996; Ohno et al., 1997; Boge et al., 1998; Berlioz-Torrent et al., 1999), also regulates Env endocytosis through AP-1 and AP-2 interactions (Wyss et al., 2001; Byland et al., 2007; Bhakta et al., 2011). In some NV17 sequences, the stop codon at position 880 was ablated and a new stop codon was generated at position 891, resulting in the addition of 11 amino acids (^880^RGTEIQSGTVY^890^) to the C terminus. While intriguing, it is not possible to state further how these changes affect the already noncanonical signal.

R722G (which flanks the ΔGY deletion) and S727P mutations have been previously associated with the loss of ΔGY-control (Fultz et al., 2001; Breed et al., 2013a; Breed et al., 2015). These mutations augment Env levels on the surface of infected cells or virions, but are not associated with new trafficking functions, nor are sufficient to restore full pathogenicity (Breed et al., 2013b; Lawrence et al., 2022). S727P has been shown to restore the ability of ΔGY to deplete gut CD4^+^ T cells in the lamina propria (Breed et al., 2013b). These mutations have been proposed as “first steps” toward loss of ΔGY-control (Lawrence et al., 2022). We observed R722G in cellular or plasma Env sequences from every animal, except NV17, and S727P in ΔGY-non-controllers (NV10, NV11). That these mutations were present in both groups again indicates that they were strongly selected for during ΔGY infection.

### Mutations in the Nef gene correlated with loss of ΔGY-control

**Figure 7.**
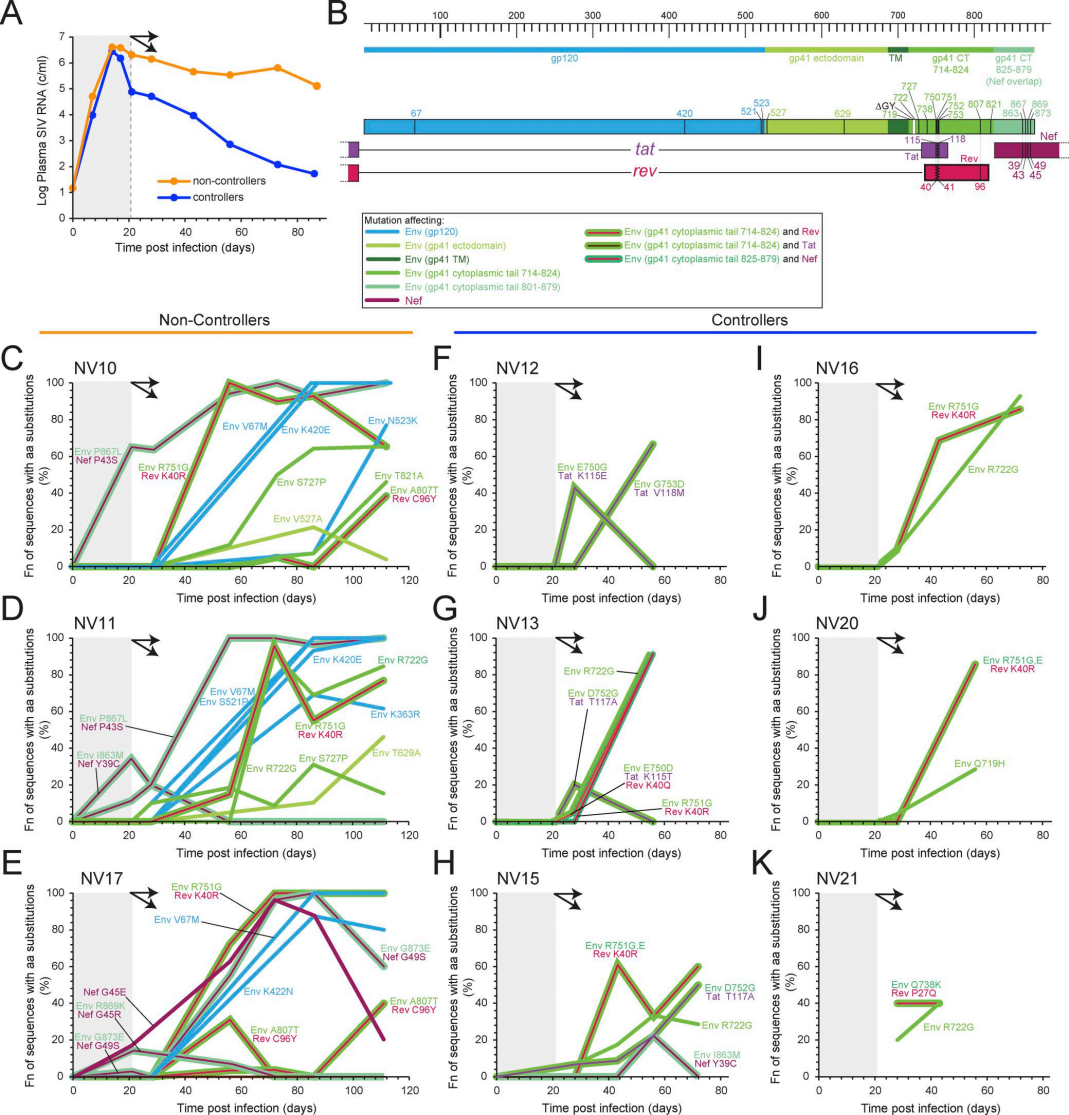
**Early Nef/Env mutations may determine elite control in ΔGY infection. (A)** Average viral loads of ΔGY-controllers and ΔGY-non-controllers. Curves diverge at day 21 after infection (double arrows). **(B)** Location of mutations. Nonsynonymous mutations (black lines) were observed in plasma virus in ΔGY-controllers and ΔGY-non-controllers. 5–50 sequences were examined per time point. Only mutations reaching a frequency (fn) of >20% at ≥1 time point(s) are shown. **(C–K)** Time course for appearance of mutations in plasma virus in ΔGY-non-controllers (C–E) and ΔGY-controllers (F–K). The shaded area indicates the 21 days before divergence of the plasma SIV RNA values (double arrows). Double lines indicate mutations affecting 2 ORFs. Only mutations reaching a frequency of >20% are shown. aa, amino acid.

Because the viral loads of the ΔGY-controllers and ΔGY-non-controllers diverged significantly by day 21, we focused our analysis on plasma virus collected during this critical period to determine whether mutations in the virus correlated with these different outcomes (Fig. 7). In the ΔGY-controllers, *env* mutations were not observed during this period. In the ΔGY-non-controllers, all mutations arising during the first 21 days of infection occurred in the Env cytoplasmic tail, in a genomic region that overlaps the *nef* ORF. While these mutations were silent or missense in regard to Env, all of the observed mutations were missense Nef mutations (Fig. 7). We hypothesize that the observed *env* mutations are operating through their effects on the *nef* coding sequence. The clustering of mutations at Nef codons 39, 43, 45, and 49, a signature unique to the ΔGY-non-controllers, suggests that these changes could represent cytotoxic T lymphocyte (CTL) escape mutations. A particularly compelling example was seen in NV17—a mutation in the second position of *env* codon 869 arose by day 21 (Fig. 7 E) and produced substitutions in both Env and Nef (Env R869K/Nef G45R). This mutation then declined in frequency, while a mutation in the third position of this same codon becomes more frequent. This further mutation was *env*-silent, but *nef*-missense (Nef G45E). Although functional effects of these changes to Env/Nef cannot be ruled out, it is possible that strong, early, Nef-specific CTL responses were involved in the immune control of ΔGY-infection, with putative Nef escape mutations corresponding to a lack of control. Interestingly, we identified potential neutralizing antibody escape mutations at position 420 in the V4 region of gp120 in the ΔGY-non-controllers, but not the ΔGY-controllers (Fig. 7). Mutations at this position have been associated with escape from autologous neutralizing antibody responses in several previous studies (Yeh et al., 2012; Ita et al., 2018; Fray et al., 2023). Importantly, this mutation became prominent only after the trajectory of non-control was evident, and is thus unlikely to be the dominant factor in the lack of control in these animals. Rather, it likely reflects the expected viral escape from an emerging autologous neutralizing antibody response.

## Discussion

The study of ECs provides valuable insight into the prospect of a functional cure for HIV-1 infection. However, elite control is exceedingly rare, and few studies have observed the early period during which control is established (Goujard et al., 2009; Okulicz et al., 2009; Madec et al., 2013; Chen et al., 2014; Walker-Sperling et al., 2017; Moosa et al., 2018; Morley et al., 2019; Kazer et al., 2020). Thus, many aspects of elite control, including its exact mechanism(s), remain a mystery. Additionally, reservoir dynamics in ECs are difficult to analyze because ECs, like those who initiate ART early in infection (Ananworanich et al., 2012; Archin et al., 2012; Jain et al., 2013; Persaud et al., 2014; Schuetz et al., 2014; Luzuriaga et al., 2015; Foster et al., 2017; Henrich et al., 2017; Okoye et al., 2018; Whitney et al., 2018; Luo et al., 2019; Bachmann et al., 2019; Martin et al., 2020; Shelton et al., 2021; Wang et al., 2022; Clain et al., 2023), have lower levels of total (Julg et al., 2010; Hatano et al., 2013a) and integrated HIV DNA (Graf et al., 2011) and smaller reservoirs than those who initiate treatment during chronic infection (Jiang et al., 2020; Kwaa et al., 2020).

Although the ΔGY-model involves a genetically modified virus, it does recapitulate some of the defining features of elite control in humans (Breed et al., 2015), and has provided, for the first time, the opportunity to quantify the dynamics of reservoir formation and maintenance in the setting of natural control. Moreover, because three of the nine animals in this study unexpectedly failed to control the virus and required ART for viral suppression, we had the opportunity to compare reservoir formation and maintenance in the settings of natural control and pharmacologic, ART-mediated control.

Following day 21, immune responses in ΔGY-controllers were able to maintain ΔGY-control at elite levels (<15 RNA copies/ml), with no viral escape. Indeed, in our collective studies of 35 ΔGY-controllers (unpublished data) (Breed et al., 2013a; Breed et al., 2013b; Breed et al., 2015), once viral load decreased to <100 copies/ml, no viral rebound occurred in any animal through 5–9 years of follow-up. That plasma virus decay was near-equally rapid for both natural and ART-mediated control indicates that natural control blocks new infection events almost as well as ART. The similar, days-long timeframe for decay of peripheral blood CD4^+^ T cells containing intact proviruses between both groups also demonstrates the effectiveness of this natural control.

It is important to note that studies of decay dynamics in HIV-1, SIV, and SHIV infection have shown that a quasi-stable third phase of infected cell decay does not become apparent until after 2 years of ART (White et al., 2022; Fray et al., 2023; Kumar et al., 2023). Prior to that time, the infected cell population is dominated by cells that do not become part of the stable reservoir. Longer term follow-up studies of ΔGY-infected animals will be required to determine the relationship between the infected cells that persist in the setting of natural and pharmacologic control and the cells that persist in PWH on long-term ART.

Consistent with previous observations (White et al., 2022), we found that the first phase of decay of cells containing intact proviruses in peripheral blood was much slower than the first-phase decay of plasma viral RNA. One explanation is that the majority of the virus-producing cells are not located in circulation, but rather are present in the secondary lymphoid tissues where T cell activation takes place. However, using serial LN biopsies, we showed that first-phase decay was nearly identical between blood and LNs for both groups of animals (Fig. 2). One possibility is that the cells producing most of the plasma virus are also not present at high frequency in the peripheral LNs and so must be localized to other tissues. However, given that ΔGY infection has been shown to spare macrophages, the CNS, and gut CD4^+^ T cells (Breed et al., 2013a), which are common sites of infection for wild-type SIVs and HIV-1 (Veazey et al., 1998; Guadalupe et al., 2003; Brenchley et al., 2004; Brenchley et al., 2006; Mehandru et al., 2004; Li et al., 2005; Li et al., 2008; Mattapallil et al., 2005; Nilsson et al., 2007; Marchetti et al., 2008; Sankaran et al., 2008; Epple et al., 2009; Jiang et al., 2009; Cassol et al., 2010; Ciccone et al., 2010; Estes et al., 2010; Nazli et al., 2010; Nowroozalizadeh et al., 2010; Chege et al., 2011; Merlini et al., 2011; Mavigner et al., 2012; Chung et al., 2014; Kristoff et al., 2014; Hao et al., 2015; Klase et al., 2015; Somsouk et al., 2015; Tincati et al., 2016; Barbian et al., 2018; Mak et al., 2021), this is unlikely. Another possibility is that cells producing high amounts of virus are present in LNs, but decay rapidly and represent only a small subpopulation of all the infected cells measured by IPDA. This hypothesis is consistent with recent demonstrations of tremendous variation in virus production from individual cells following antigen-driven latency reversal (Moskovljevic et al., 2024).

That essentially no viral evolution was observed in the ΔGY-controllers speaks to the early timing and strength of control. The controller animals have patterns of diversity and evolution (or lack thereof) that are strikingly similar to NHPs started on ART only 10 days after infection (Fig. 5) (Immonen et al., 2024). In contrast, phylogenetic trees from ΔGY-non-controllers showed considerable branching and changes consistent with viral replication and evolution prior to the onset of ART (Fig. 6).

The determinants of elite control in our model remain a critical issue. The fate of an animal as a controller or non-controller is established early. Within 21 days after infection, the plasma SIV RNA levels of the two groups of animals diverged (Fig. 1 C), and the earliest decay dynamics were significantly different between the two groups (Figs. 2 and 3). In the three ΔGY-non-controllers, during the proposed critical window, mutations arose in the distal Env cytoplasmic tail that were also missense in the *nef* ORF (Fig. 7). Similar changes were absent in the six ΔGY-controllers. Previous studies have identified compensatory mutations in the membrane-proximal region of the cytoplasmic tail of gp41 in RMs and PTMs that failed to control ΔGY, some of which restored trafficking functions ablated by the ΔGY mutation (Breed et al., 2013a; Breed et al., 2013b; Lawrence et al., 2022). We detected similar mutations in some animals, but only after day 21. That the early gp41 mutations failed to generate a recognizable trafficking signal raises the possibility that early mutations in Nef, rather than Env, were being selected for. Such selection for one of two overlapping ORFs has been observed previously (Hughes et al., 2001). These findings are consistent with early viral escape from Nef-restricted immune responses, which conferred a growth advantage to ΔGY that resulted in lack-of-control and sustained viremia. In ΔGY-controllers, cellular responses were sufficiently potent and/or optimally timed to prevent viral escape. Alternatively, such mutations may have improved viral fitness through effects related to Env trafficking or enhanced Nef function, including the immune evasion functions of Nef (Kirchhoff et al., 2008; Pawlak and Dikeakos, 2015; Buffalo et al., 2019; Joas et al., 2020). While further studies will be required to probe the cellular immune responses to Nef among ΔGY-controllers and ΔGY-non-controllers, these findings suggest that the timing and potency of host immune responses, and the early acquisition of escape mutations, are key to determination of long-term control. Although early events in the establishment of elite control of HIV-1 are difficult to observe, our results are consistent with recent studies of immune escape mutations and HIV-1 integration sites in ECs. Lian et al. have proposed that immune pressure can eliminate infected cells before viral escape mutations are selected, resulting in a reservoir of intact proviruses with relatively few mutations (Lian et al., 2021). The subset of these proviruses that are integrated into transcriptionally unfavorable genomic regions is able to persist, giving rise to the unique integration site profile observed in ECs (Jiang et al., 2020; Lian et al., 2021).

In summary, the ΔGY-model of natural SIV control provided the opportunity to compare the dynamics of reservoir formation and maintenance during natural and pharmacologic control. We found that natural control was nearly as effective as ART in blocking new infections and viral evolution. The rapid early decay of plasma virus was not reflected in either PBMCs or LNs, suggesting most of the plasma virus is produced by only a small fraction of cells. While CD8^+^ cellular responses, and not neutralizing antibodies, have been implicated in mediating ΔGY-control (Breed et al., 2015), it remains unclear whether CTL-, NK-, and/or antibody-dependent cellular responses are involved. ΔGY-infection is the first highly reproducible NHP model of natural control and is well suited for future studies evaluating immune and/or pharmacologic interventions that can impact the size and/or the replicative capacity of the latent reservoir.

### Limitations of the study

Because of the extended period of time required to observe dynamics of the stable latent reservoir (>2 years) (White et al., 2022; Fray et al., 2023; Kumar et al., 2023), we only observed the initial two phases of decay following immunologic or pharmacologic control in our 66-wk study.

It is also important to note that our observations on the natural and pharmacologic control of ΔGY-infection are based on a relatively small number of animals and therefore must be interpreted with caution. Particularly problematic is the small number of non-controllers, which is a reflection of the fact that rapid control of viral replication is the typical outcome of ΔGY infection of PTMs. In comparable infection studies, ΔGY was controlled in 29 of 32 PTMs (91%) inoculated at 100 TCID_50_, 7 of 10 PTMs (70%) inoculated with 2000 TCID_50_ (including the present study), and 2 of 5 PTMs inoculated with 20,000 TCID_50_ (40%) (unpublished data) (Breed et al., 2015). This opens the possibility that low infectious-dose inoculation is a factor in control.

PTMs do not express a functional TRIM5α protein (Liao et al., 2007; Brennan et al., 2007; Brennan et al., 2008; Newman et al., 2008), have higher levels of basal immune activation (Klatt et al., 2010; Klatt et al., 2012; Canary et al., 2013), and typically exhibit more rapid disease progression than the RMs that are used in most NHP studies (Goldstein et al., 2005; Polacino et al., 2008; Klatt et al., 2012). It is possible that some of our observations reflect intrinsic features of the combination of virus and host.

Given that the three ΔGY-non-controllers share both an A (A019g2) and B (B016b) haplotype (Table 1), it is tempting to ascribe the lack of control to the presence of these alleles. However, in previous cohorts of ΔGY infected PTMs, these alleles have been present in ΔGY-controllers (unpublished data) (Breed et al., 2015). The Mane-A1*084 (formerly Mane-A*10) allele has been associated with neuroprotection (Mankowski et al., 2008; Beck et al., 2016) and strong, immunodominant CD8^+^ T cell responses (Smith et al., 2005b), but typically does not result in viral control (Fernandez et al., 2005; Smith et al., 2005a; Smith et al., 2005b; Loh et al., 2007; Loh et al., 2008; Beck et al., 2016). Rather, just as HLA-B*57 and HLA-B*27 in humans (Fellay et al., 2007; Dalmasso et al., 2008; Fellay et al., 2009; Limou et al., 2009; International HIV Controllers Study et al., 2010; Pelak et al., 2010; Carrington and Walker, 2012; McLaren et al., 2012; McLaren et al., 2015; McLaren et al., 2017) and Mamu-B*08 and Mamu-B*17 in RMs (O’Connor et al., 2003; Loffredo et al., 2006; Loffredo et al., 2007; Yant et al., 2006; Mee et al., 2009; Aarnink et al., 2011; Budde et al., 2012; Mudd et al., 2012; Passaes et al., 2020) are neither necessary nor sufficient for control, in this model host genetics alone cannot adequately account for the control phenotype. In RMs, there is evidence that some MHC alleles, alone or in association with specific KIR genes, can confer more rapid progression to AIDS (Albrecht et al., 2014), but little evidence for PTM alleles that confer the same (Mankowski et al., 2008; Gooneratne et al., 2014; Beck et al., 2016). The PTM MHC is the least well defined of those of the main laboratory NHP models (Lafont et al., 2003; Smith et al., 2005a; Pratt et al., 2006; Loh and Kent, 2008; Fernandez et al., 2011; Gooneratne et al., 2014; Roodgar et al., 2020).

## Materials and methods

### Experimental model and subject details

PTMs (*M. nemestrina*) used in this study were purpose-bred at Johns Hopkins University and moved to Tulane National Biomedical Research Center for this project. This study was reviewed and approved by the Institutional Animal Care and Use Committee of Tulane University. Animals were cared for in accordance with the NIH’s Guide for the Care and Use of Laboratory Animals. Procedures for handling and ABSL2 containment of animals were approved by the Tulane University Institutional Biosafety Committee. The Tulane National Biomedical Research Center is fully accredited by the Association for Accreditation of Laboratory Animal Care. Animals were infected intravenously with 2000 TCID_50_ barcoded SIVmac239ΔGY virus. Cohort details are described in Table 1.

### Antiretroviral therapy

At week 16 after infection, animals NV10, NV11, and NV17 initiated an ART regimen of tenofovir (PMPA) 20 mg/kg/day, emtricitabine (FTC) 30 mg/kg/day, and dolutegravir (DTG) 2.5 mg/kg/day, dissolved in KLEPTOSE and delivered subcutaneously. At week 38, the regimen was changed to tenofovir (TDF) 5 mg/kg/day, emtricitabine (FTC) 30 mg/kg/day, and dolutegravir (DTG) 2.5 mg/kg/day (Del Prete et al., 2016).

### Animal genotyping

Genomic DNA was isolated from whole blood samples with a Maxwell RSC 48 instrument using a Maxwell RSC Buffy Coat DNA kit according to the manufacturer’s protocol (Promega). Amplicons for deep sequencing were generated as described previously (Shortreed et al., 2020). Briefly, a panel of oligonucleotides flanking the highly polymorphic peptide domains encoded by exon 2 of MHC class I and class II loci were used for multiplex PCRs with an Access Array 48.48 (Standard BioTools) following the manufacturer’s protocol. This amplicon library was sequenced on an Illumina MiSeq instrument, and the resulting sequence reads were mapped against a custom reference database of *Mane* MHC sequences (https://github.com/dholab/mhc_genotyper). Mane haplotypes were inferred based on the combinations of *Mane* class I and class II allelic variants identified in each animal, as described previously (Semler et al., 2018).

### Viral loads

Plasma SIV RNA levels were determined using a *gag*-targeted quantitative real-time/digital RT-PCR assay as previously described, with six replicate reactions analyzed per extracted sample for an assay threshold of 15 SIV RNA copies/ml (Li et al., 2016). Samples that did not yield any positive results across the replicate reactions were reported as a value of “less than” the value that would apply for one positive reaction out of six (Li et al., 2016).

### DNA extraction

PBMCs were obtained from whole blood by layering on Lymphoprep media (StemCell Technologies) and centrifuging to isolate PBMCs. PBMCs were washed, counted, and cryopreserved at 5–10 × 10^6^ cells/ml in Bambanker media (Bulldog-Bio). Peripheral LNs (axillary or inguinal) were obtained by excisional biopsy and processed to single-cell suspension by dicing with a scalpel, followed by pushing through a 70-μm cell strainer. Cells were washed, counted, and cryopreserved at 5–10 × 10^6^ cells/ml in Bambanker media (Bulldog-Bio). The cryopreserved PBMCs or LN cells were rapidly thawed in RPMI + 50% heat-inactivated fetal bovine serum. Cells were aliquoted, with some devoted to characterization by flow cytometry, while the rest were lysed and DNA was extracted using the QIAamp DNA mini kit (Qiagen), according to the manufacturer’s instructions.

### Flow cytometry

**Figure S1.**
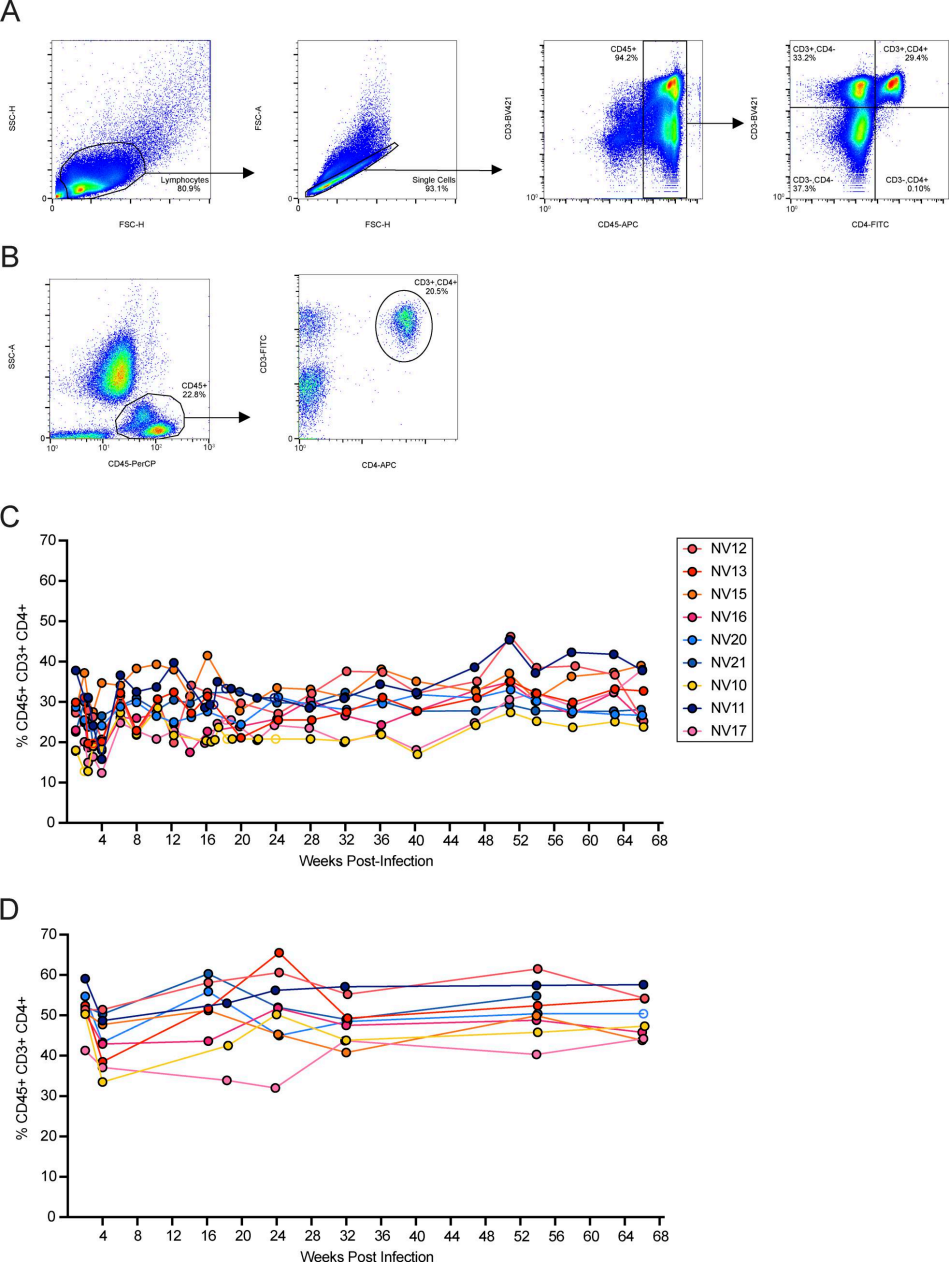
**Representative gating strategy and CD4** ^**+**^ **T cell counts. (A and B)** Gating strategy for determining the percentage of CD3^+^CD4^+^ T cells among CD45^+^ cells in PBMCs (A) and in whole blood samples (B). **(C and D)** Longitudinal analysis of CD4^+^ T cell counts in PBMCs (C) and LNs (D). Open circles represent rare time points for which flow cytometric quantification was unavailable. Due to the relative stability of the CD4^+^ population and close spacing of time points, we used the value closest to the missing value in date for the normalization calculation.

Because of limited blood volumes that could be safely obtained from the small animals in the study, and thus limited number of cells acquired, we extracted DNA for the IPDA from total PBMCs, rather than from purified CD4^+^ T cells as is typical (Bruner et al., 2019; Bender et al., 2019; Fray et al., 2023). Analysis of cell populations other than purified CD4^+^ T cells is not unprecedented; for example, clinical CD4^+^ T cell counts can be used to adjust IPDA data collected from DNA extracted from whole blood (Dragoni et al., 2022). To report the data as “per million CD4^+^ T cells,” we corrected the IPDA measurements using the percentage of CD3^+^,CD4^+^ of CD45^+^ cells. Cells were stained at 4°C with BV421 mouse anti-human CD3 clone SP34-2, FITC mouse anti-human CD4 clone L200, APC mouse anti-NHP CD45 clone D058-1283 (BD Biosciences), and Fixable Viability Dye eFluor 780 (Thermo Fisher Scientific) for 30–45 min. This panel was validated with appropriate isotype and single-color controls. After washing the cells, data were collected on an Intellicyt flow cytometer and analyzed using FlowJo software (TreeStar). A representative gating strategy is shown in Fig. S1 A. For some samples, CD4^+^ T cell percent was monitored by flow cytometric evaluation of absolute counts. 50 μl of whole blood was stained with a four-color panel of FITC-CD3 clone SP34, PerCP-CD45 clone D058-1283, APC-CD4 clone L200, and V500-CD8 clone SK1 (BD Biosciences). Samples were incubated for 20 min at room temperature in the dark at the recommended volume. Red blood cells were lysed with 450 μl of 1X BD FACS Lysing Solution for 30–45 min. The sample was both mixed and volumetrically analyzed on a Miltenyi MACSQuant 16. A representative gating strategy is shown in Fig. S1 B. CD4^+^ T cell counts in PBMCs (Fig. S1 C) and LNs (Fig. S1 D) were relatively stable throughout the study.

### Droplet digital PCR

All primer and probe sequences for droplet digital PCR (ddPCR) are included in Table S3. Conditions specific to each assay are described under each subheading. Droplets were made with Bio-Rad QX200 Automated Droplet Generator. All thermocycling was performed using the updated conditions described by Fray et al. (2023): 95°C for 10 min, followed by 50 cycles of (95°C for 30 s, 56°C for 2 min), and a final hold of 98°C for 10 min, with a ramp rate for all steps of 2°C/s, performed in a Bio-Rad C1000 Touch thermal cycler. Droplets were read by a Bio-Rad QX200 Reader and analyzed with QX Manager (formerly QuantaSoft Analysis Pro) using the legacy droplet volume of 0.85 nl for consistency before and after software update and for direct comparison with published datasets. Wells with fewer than 10,000 droplets were not analyzed. All reaction volumes were 22 μl.

### RPP30

The RPP30 ddPCR assay was used to calculate a DNA shearing index and normalize data to cell input and was performed as described previously (Fray et al., 2023) using primers and probes developed by Bender and colleagues (Bender et al., 2019). Primers were used at a concentration of 500 nM and probes at a concentration of 250 nM. The input DNA was typically diluted 150-fold (ideally for an input of ≤2 ng DNA/reaction), but was adjusted depending on the sample. We used 5.5 μl of diluted DNA as input.

Because this is, to our knowledge, the first published report using the SIV IPDA in the PTM, we confirmed that the RPP30 primer/probes and amplicon distance were conserved in the PTM. The primer/probe sequences are conserved, and the difference in distance between the amplicons (*M. nemestrina* 1,866 bp and *Macaca mulatta* 1,870 bp) is negligible for the purposes of this assay (1,627 bp apart in SIVmac239). The Genome Institute at Washington University School of Medicine Mmul_10 version of the RM genome (also called rheMac10) and the Baylor College of Medicine Mnem_1.0 version of the PTM genome were downloaded from the UCSC Genome Browser (http://genome.ucsc.edu) (RRID:SCR_005780) (Perez et al., 2025) for this analysis.

### IPDA

The IPDA ddPCR was performed as described with minor modifications (Bender et al., 2019). We performed five replicates per time point. We used 8.5 μl of DNA as input, ideally adding 200–700 ng of DNA to each reaction. We used a different reverse primer for the *env* amplicon that is specific to the SIVmac239 consensus sequence. Primers were used at a working concentration of 600 nM and probes at a working concentration of 200 nM.

### env-2LTRc

The *env*-2LTRc ddPCR assay was performed as described previously (Bender et al., 2019; Fray et al., 2023) as a duplex of an amplicon spanning the 2LTR junction (Policicchio et al., 2018) and the IPDA *env* amplicon. We performed three replicates per time point and used the SIVmac239-specific primer in the duplexed *env* reaction. We used 5.5 μl of DNA as input. Primers were used at a working concentration of 600 nM and probes at a working concentration of 200 nM.

### HPDA

The HPDA ddPCR assay was performed as described previously (Fray et al., 2023). We performed six replicates per time point and used the SIVmac239-specific primer. We used 5.5 μl of DNA as input. Primers were used at a working concentration of 600 nM and probes at a working concentration of 200 nM.

### Modeling of decay processes

We used a nonlinear mixed-effects approach to fit models to the decay of viral load and SIV DNA species in the six ΔGY-controllers and the three ΔGY-non-controllers, in blood and LNs. In both groups and for all SIV DNA species, the decay was evaluated from peak viral load in plasma and the first data point for LNs.

For controller animals, we fitted a biphasic decay model that is given by

$$Y=Y_{0}\left(Ae^{a_{1}t}+\left(1-A\right)e^{a_{2}t}\right)$$ (1)

where Y is the variable of interest, $Y_{0}$ is the baseline value, A is the fraction of Y that decays in the first phase with decay rate $a_{1}$, and (1−A) is the fraction of Y, which decays in the second phase with decay rate $a_{2}$.

For the non-controller macaques, we fitted a combination of single and biphasic decay models that are given by

$$Y=\left\{ \begin{array}{l} Y_{0}e^{a_{3}t}\;\;\;\;\;\;\;\;\;\;\;\;\;\;\;\;\;\;\;\;\;\;\;\;\;\;\;\;\;\;\;\;\;\;\;\;\;\;\;\;\;\;\;\;\;\;\;\;\;\;\;\;\;\;\;\;\;\;\;\;\;\;\;\;\;\;\;if\;t<t_{T}\\ Y_{0}e^{a_{3}t_{T}}\;\left(Be^{b_{1}\left(t-t_{T}\right)}+\left[1-B\right]e^{b_{2}\left(t-t_{T}\right)}\right)\;if\;t\geq t_{T} \end{array}\right..$$ (2)

The single decay model was fit to SIV DNA from post-peak viral load to ART initiation $t_{T}$, while the biphasic decay model was fit to SIV DNA following ART initiation. Note that Eqs. 1 and 2 were fitted simultaneously to controller and non-controller macaques, where the baseline value $Y_{0}$ and $a_{1}$/$a_{3}$ can be shared (i.e., equal) assuming that the post-peak viral load decay is the same for both animal groups until ART is initiated. ART leads to a biphasic decay as shown before (Ho et al., 1995; Wei et al., 1995; Perelson et al., 1996; Perelson et al., 1997). This ART-associated biphasic decay consists of B, the fraction of Y that decays in the first phase following ART initiation with decay rate $b_{1}$, and (1−B) is the fraction of Y, which decays in the second phase with decay rate $b_{2}$.

With this general model, we tested multiple assumptions:

(1) All phases, except $a_{1}=a_{3}$, are different for both groups: $a_{2}\neq b_{1}\neq b_{2}$.
(2) The post-peak viral load decay continues for ΔGY-non-controllers under ART: $a_{3}=b_{1}$ and $a_{2}\neq b_{2}$.
(3) The final decay phases are the same for both groups: $a_{1}=a_{3}\neq b_{1}$ and $a_{2}=b_{2}$.
(4) The early decay in ΔGY-controllers and pre-ART decay in ΔGY-non-controllers are different, with acceleration of the decay after ART, but the long-term decay under ART is the same as the second-phase decay in ΔGY-controllers: $a_{1}\neq a_{3}\neq b_{1}$ and $a_{2}=b_{2}$.

To fit Eqs. 1 and 2 to the data, we used a nonlinear mixed-effects model, which assumes that a model parameter for individual i is given by $\theta_{i}=\theta e^{\beta +\eta_{i}}$ with $\eta_{i}=N\left(0,\omega_{i}^{2}\right)$, with θ being the median value of the population parameter, β being a potential covariate (for ΔGY-controller vs. ΔGY-non-controller), and η being the normally distributed random effect (individual parameter) (Lavielle, 2014). Models were fit using the software Monolix 2024R1 (Lixoft). Formally, to test the models above, we included a covariate (for ΔGY-controller versus ΔGY-non-controller) for parameters $a_{1}$/$a_{3}$ and $a_{2}$/$b_{2}$ (e.g., $a_{1}$ = $a_{3}$ if the covariate is β = 0). This approach tests whether the data provide support for different rates of decay or not for ΔGY-controllers versus ΔGY-non-controllers. We statistically compared the quality of the model fits using the corrected Bayesian information criterion (Burnham and Anderson, 2002). 95% CIs of model parameters were calculated with Monolix, and the significance of the covariate effect was tested using the Wald test in Monolix. Wilcoxon’s tests were applied to evaluate differences in decay rates across decay phases (paired test), as well as between ΔGY-controller and ΔGY-non-controller animals (unpaired test).

For all DNA species studied, the best model (or equivalent best) was model 4, where the early decay in ΔGY-controllers and pre-ART decay in ΔGY-non-controllers are different, with acceleration of the decay after ART, but the long-term decay under ART is the same as the second-phase decay in ΔGY-controllers (i.e., $a_{1}\neq a_{3}\neq b_{1}$ and $a_{2}=b_{2}$).

To evaluate uncertainty in both individual and population predictions, we included 95% confidence bands in all relevant model visualizations. For individual-level predictions, we accounted for parameter uncertainty by generating 1,000 simulated parameter sets per subject using Monolix. These sets were drawn from the individual posterior distributions estimated during the model fitting process. Each parameter set was used to simulate a full individual profile, producing 1,000 predicted trajectories per subject. From these, we constructed pointwise 95% CIs by extracting the 2.5th and 97.5th percentiles at each time point. This method captures the uncertainty stemming from individual parameter estimation.

At the population level, we employed Simulx (Lixoft) to conduct a clinical trial simulation consisting of 1,000 virtual individuals over 100 replicates. The simulations used fixed, typical population parameters, excluding any interindividual variability (i.e., random effects were not applied). This design isolates uncertainty attributable to residual variability and model structure, independent of between-subject variation. From these simulations, we computed 95% confidence bands at each time point by calculating the relevant percentiles across the replicates.

The 95% CIs for individual parameters were calculated based on the Monolix estimated mode plus and minus 1.96 times the standard deviation for each parameter, which assumes an approximate normal distribution after the appropriate transformation (e.g., log-normal or logit-normal).

### Envelope sequencing and analysis

DNA used for ddPCR was also used for this analysis, carried out by limiting dilution nested PCR. ddPCR data were used to estimate the concentration of proviruses in each sample, which was used to determine the appropriate dilution such that ≤30% of reactions were positive, and thus by Poisson’s statistics, there is a ≥80% chance each positive reaction resulted from the amplification of a single provirus (Hiener et al., 2017; Lee, 2021). Samples were diluted in 10 mM Tris-HCl. The PCR was designed to encompass the entire *env* gene, as well as the barcode (inner PCR amplicon = 3,520 bp), which sits between *vpx* and *vpr*. All primer sequences are included in Table S3. Both the outer and inner PCRs were 20 μl reactions using the following working concentrations of reagents: 1X high fidelity buffer, 2 mM MgSO_4_, 0.2 mM dNTPs, 200 nM each primer, and 0.025 units/μl Platinum Taq DNA Polymerase High Fidelity (Thermo Fisher Scientific). The outer PCR used 2 μl of DNA as a template. Cycling conditions were 94°C for 2 min, followed by 35 cycles of (94°C for 30 s, 55°C for 30 s, 72°C for 4 min), and a final hold of 72°C for 10 min (Breed et al., 2013a). The outer PCR was diluted twofold with 10 mM Tris-HCl, and 1 μl of diluted product was used as the template for the inner PCR. Cycling conditions for the inner PCR were 94°C for 2 min, 45 cycles of (94°C for 30 s, 55°C for 30 s, 72°C for 4 min), and a final hold of 72°C for 4 min (Breed et al., 2013a). 2 μl of inner PCR product was added to 28 μl of loading dye, run on a 1% agarose gel, and visualized with ultraviolet illumination. Positive wells were diluted fivefold with 10 mM Tris-HCl and sent for Sanger sequencing (Azenta). Primers used for sequencing were those used for the inner PCR, as well as additional primers in Table S3. Chromatograms were trimmed and assembled to the SIVmac239 genome using the “Map to Reference” function in Geneious Prime (Dotmatics). Chromatograms were scanned by hand to resolve ambiguous base calls and look for overlapping peaks indicative of multiple templates in the same well or Taq-induced PCR errors.

### Full-length sequencing and analysis

As before, ddPCR data were used to estimate the concentration of proviruses in each sample, which was used to determine the appropriate dilution factor to reach the limiting dilution by Poisson’s statistics. Samples were diluted in 1X PBS. To perform whole genome amplification (WGA), we used the REPLI-g Advanced Single Cell DNA kit (Qiagen), with several modifications. Namely, we followed the manufacturer’s protocol for WGA from single cells in 96 wells, but we used 1 μl of our DNA dilution as the template, and used half the recommended volume of all other reagents: 0.75 μl buffer D2 (for 96 reactions, made from 6.5 μl 1 M DTT and 71.5 μl reconstituted DLB), 0.75 μl stop solution, and 10 μl of master mix (for 96 reactions, made from 225 μl H_2_O sc, 725 μl REPLI-g reaction buffer, and 50 μl REPLI-g sc polymerase), for a final reaction volume of 12.5 μl. We found that these small volumes required us to centrifuge the plate at greater than the recommended 1,000 rpm during all mix and spin-down steps. We incubated the reaction for 3 h at 30°C in a thermocycler with the lid set to 70°C. The final product was diluted 25-fold with 10 mM Tris-HCl.

The WGA product was used as the template for four nested-PCRs that yield overlapping amplicons that tile the full genome of SIVmac239, a scheme we named Sequencing of Macaque-Integrated Lentiviruses (SMILe). The primers for each reaction can be found in Table S3. Both the outer and inner PCRs were 20 μl reactions using 2 μl of diluted WGA product and the following working concentrations of reagents: 1X high fidelity buffer, 2 mM MgSO_4_, 0.2 mM dNTPs, 400 nM each primer, and 0.05 U/μl Platinum Taq DNA polymerase High Fidelity (Thermo Fisher Scientific). Both the outer and inner PCRs used the following cycling conditions: 94°C for 2 min, 10 cycles of (94°C for 15 s, a touchdown step beginning at 65°C and decreasing at 1°C/cycle for 30 s, 72°C for 3 min), followed by 25 cycles of (94°C for 30 s, 55°C for 30 s, 72°C for 4 min) and a final hold of 72°C for 7 min. The outer PCR was diluted twofold with 10 mM Tris-HCl before 2 μl was used as the template for the inner PCR. 2 μl of inner PCR product was added to 28 μl of loading dye, run on a 1% agarose gel, and visualized with ultraviolet illumination. Wells for which all four PCRs yielded a positive result were presumed “intact” and selected for sequencing. These wells were diluted fivefold with 10 mM Tris-HCl and sent for Sanger sequencing (Azenta). Primers used for sequencing were those used for the inner PCR, as well as additional primers in Table S3. Chromatograms were analyzed and inspected as before.

### Plasma virus sequencing

Plasma samples were thawed and aliquoted based on viral load, such that ∼10,000 copies of SIV RNA were used in each reaction. Viral RNA was extracted as previously described (Tosiano et al., 2019). Briefly, plasma aliquots were spun for 15 min at 2,700 *g* at 4°C to pellet debris. The supernatant was transferred to clean 1.5-ml tubes and spun at 21,000 *g* for 2 h at 4°C to pellet virions. The supernatant was removed, and virions were lysed by adding 100 μl of 3 M guanidinium hydrochloride supplemented with 50 mM Tris-HCl, 1 mM CaCl_2_, and fresh 1 mg/ml proteinase K. Samples were placed at 42°C for 1 h, vortexing every 20 min. 400 μl of 6 M guanidinium thiocyanate supplemented with 50 mM Tris-HCl, 1 mM EDTA, and 600 μg/ml of glycogen was added, and samples were mixed, followed by the addition of 500 μl room-temperature isopropanol and vortexing. The samples were incubated at −80°C overnight. The following day, samples were centrifuged at 21,000 *g* for 10 min at room temperature. The supernatant was discarded, and the pellet was washed with 500 μl 70% ethanol. Samples were centrifuged at 21,000 *g* for 10 min at room temperature. Ethanol was removed, and the RNA pellet was dried for 10 min. RNA was resuspended in 20 μl 5 mM Tris-HCl and immediately used to generate cDNA. A mixture of 20 μl RNA was mixed with a working concentration of 0.5 mM dNTPs and 100 nM of the outer reverse primer in a final volume of 25 μl. The mixture was incubated in a thermocycler at 65°C for 10 min and then placed in a metal plate holder that was precooled at −20°C. To this initial mixture, we added a 25 μl mixture of working concentrations of 1X first-strand buffer, 1 mM DTT, 1 mM RNaseOUT, and 4 U/μl SuperScript III reverse transcriptase (Thermo Fisher Scientific). The plate was returned to the thermocycler and incubated at 50°C for 50 min, followed by 85°C for 10 min. The resulting cDNA was serially diluted to determine the limiting dilution and amplified as described before.

### Bioinformatics of sequencing analysis

Because sequence diversity was so low, and length permutations were rare, we aligned sequences by hand in MEGA7 (Kumar et al., 2016). Full-length sequences collected by SMILe were analyzed using the Los Alamos National Laboratory HIV sequences database Gene Cutter tool and determined to be intact or defective using the criteria outlined by Hiener and colleagues (Hiener et al., 2018). We included sequences in downstream analysis that were defective in regions outside of *env*. All *env* provirus and plasma virus sequences were also subject to Gene Cutter analysis to identify defects in the *env* gene. All sequences were also analyzed using the Los Alamos National Laboratory HIV sequences database Hypermut tool (Rose and Korber, 2000) to identify hypermutated sequences. The Los Alamos National Laboratory HIV sequences database ElimDupes tool was used to identify and collapse identical sequences. Neighbor-joining trees were generated in MEGA7. P-distances were calculated using the maximum composite likelihood model in MEGA7 (Kumar et al., 2016). Trees were rooted to the ΔGY inoculum sequence.

### Quantification and statistical analysis

Details regarding the statistical tests used and the represented data can be found in the figure legends, or, for the mathematical modeling, in the “Modeling of decay processes section.” Statistical tests were considered significant when P values were <0.05. Statistical analyses were performed in GraphPad Prism 10 (Prism Software) or, for the modeling of decay processes, Monolix 2020R1 (Lixoft).

### Online supplemental material

Fig. S1 shows a representative gating strategy for normalizing ddPCR data from total PBMCs or whole blood, and CD4^+^ T cell counts. Table S1 provides the decay parameters for animal groups (controllers and non-controllers) from peripheral blood and LNs. Table S2 provides the decay parameters for individual animals from peripheral blood and LNs. Table S3 lists primers and probes used in this manuscript.

## Supplementary Material

Table S1 shows decay parameters for animal groups from peripheral blood and LNs.

Table S2 shows decay parameters for individual animals from peripheral blood and LNs.

Table S3 shows primer/probe details.

## Data Availability

Sequences generated in this study have been deposited in GenBank (PX476306-PX480007) and are publicly available as of the date of publication.
