# Supplementary material for: Dynamics of natural and pharmacologic control of an SIV variant with an envelope trafficking defect
Source: J Exp Med. 2025 Dec 5;223(2):e20251172. doi: 10.1084/jem.20251172 (PMC12679998; doi:10.1084/jem.20251172)
Supplement: Table S1 — shows decay parameters for animal groups from peripheral blood and LNs. [file jem_20251172_tables1.docx]

| Decay Parameters from Peripheral Blood/Plasma | | | | | | | | | |
| --- | --- | --- | --- | --- | --- | --- | --- | --- | --- |
|  | $Y$ | $Y_{0}$  (log_10_) | $A$ (ctrl)  or  $B$ (non-ctrl) | $a_{1}$(ctrl)  or  $a_{3}$ (non-ctrl)  (/day) | 1^st^ phase *t_1/2_* (ctrl)  or  pre-ART *t_1/2_*  (non-ctrl)  (days) | $b_{1}$  (/day) | 1^st^ phase  post-ART *t_1/2_*  (days) | $a_{2}$ (ctrl)  or  $b_{2}$ (non-ctrl)  (/day) | 2^nd^ phase *t_1/2_* or T_d_ (days) |
| Controllers | VL | 5.96  [5.56, 6.37] | 0.99  [0.99, 0.99] | -0.17  [-0.21, -0.14] | 4  [3.3, 5.0] | N/A | N/A | -0.0087  [-0.017, -0.00091] | 80  [40.8 –762] |
|  | Intact | 4.22  [3.96, 4.49] | 0.83  [0.75, 0.90] | -0.034  [-0.0380, -0.031] | 20.4  [18.2, 22.4] | N/A | N/A | -0.0066  [-0.0075, -0.0056] | 105  [92, 124] |
|  | HM | 2.72  [2.44, 3.01] | 0.75  [0.60, 0.89] | -0.033  [not identified] | 21.0  [not identified] | N/A | N/A | -0.00063  [-0.0021– +0.00083] | 1100  [330, +835] |
|  | 2LTR  Total | 3.68  [3.42, 3.95] | 0.83  [0.73, 0.91] | -0.026  [-0.042, -0.0096] | 26.7  [16.5, 72.2] | N/A | N/A | -0.0047  [-0.0067, -0.0026] | 148  [104 , 267] |
|  | 2LTR *env*+ | 3.62  [3.37, 3.87] | 0.80  [0.65, 0.92] | -0.028  [-0.037, -0.019] | 24.8  [19, 37] | N/A | N/A | -0.0054  [-0.0068, -0.0040] | 128  [102, 173] |
|  | 2LTR *env*- | 2.75  [2.54, 2.96] | 0.86  [0.76, 0.92] | -0.020  [-0.047, +0.0061] | 34.7  [14.7, +114] | N/A | N/A | -0.0024  [-0.0032, -0.0016] | 289  [217, 433] |
| Non-controllers | VL | 6.46  [5.82, 7.10] | 0.99  [0.99, 0.99] | -0.04  [-0.075, -0.0042] | 17  [9.2, 165] | -0.68  [-0.92, -0.43] | 1  [0.8, 1.6] | -0.0087  [-0.017, -0.00091] | 80  [40.8, 762] |
|  | Intact | 4.76  [4.37, 5.15] | 0.32  [not identified] | -0.026  [-0.030, -0.022] | 26.7  [23.1, 31.5] | -0.057  [-0.161, +0.046] | 12.2  [4.3, +15.1] | -0.0066  [-0.0075, -0.0056] | 105  [92, 124] |
|  | HM | 2.72  [2.44, 3.01] | 0.99  [0, 0.99] | -0.015  [not identified] | 46.2  [not identified] | -0.0019  [-0.0034, -0.000058] | 365  [204, 11951] | -0.00063  [-0.0021– +0.00083] | 1100  [330, +835] |
|  | 2LTR  Total | 3.68  [3.42, 3.95] | 0.55  [0.30, 0.78] | -0.011  [-0.02, -0.0023] | 63.0  [34.7, 301] | -5.0  [not identified] | 0.1  [not identified] | -0.0047 /day  [-0.0067, -0.0026] | 148  [104, 267] |
|  | 2LTR *env*+ | 3.62  [3.37, 3.87] | 0.61  [0.38, 0.80] | -0.0098  [not identified] | 70.7  [not identified] | -1.04  [-3.57, -1.49] | 0.7  [-0.2, -0.5] | -0.0054  [-0.0068, -0.0040] | 128  [102, 173] |
|  | 2LTR *env*- | 2.75  [2.54, 2.96] | 0.69  [not identified] | -0.0056  [-0.011, -0.00029] | 124  [63, 2390] | -1.72  [-9.35, +5.9] | 0.4  [0.07, +0.1] | -0.0024  [-0.0032, -0.0016] | 289  [217, 433] |

**Table S1: Decay Parameters for Animal Groups from Peripheral Blood and Lymph Nodes**

| Decay Parameters from Lymph Nodes | | | | | | | | | |
| --- | --- | --- | --- | --- | --- | --- | --- | --- | --- |
|  | $Y$ | $Y_{0}$  (log_10_) | $A$ (ctrl)  or  $B$ (non-ctrl) | $a_{1}$ (ctrl)  or  $a_{3}$ (non-ctrl)  (/day) | 1^st^ phase *t_1/2_* (ctrl)  or  pre-ART *t_1/2_*  (non-ctrl)  (days) | $b_{1}$  (/day) | 1^st^ phase  post-ART *t_1/2_*  (days) | $a_{2}$ (ctrl)  or  $b_{2}$ (non-ctrl)  (/day) | 2^nd^ phase *t_1/2_* or *T_d_* (days) |
| Controllers | Intact | 4.54  [4.19, 4.89] | 0.92  [not identified] | -0.038  [-0.092, -0.016] | 18.2  [7.5, 43.3] | N/A | N/A | -0.0038  [-0.0051, -0.0027] | 182  [136, 257] |
|  | HM | 3.86  [3.01, 4.72] | 0.99  [0.96, 0.99] | -0.18  [-0.37, -0.086] | 3.9  [1.9, 8.1] | N/A | N/A | -0.00032  [-5.16, +9.6e-09] | 2166  [0.1, +7.2e+7] |
|  | 2LTR  Total | 3.61  [3.33, 3.89] | 0.94  [0.77, 0.99] | -0.012  [-0.018, -0.0084] | 57.8  [38.5, 82.5] | N/A | N/A | -0.00032  [-0.89, -1e-7] | 2166  [0.8, 6.9e+6] |
|  | 2LTR *env*+ | 3.58  [3.25, 3.91] | 0.95  [0.88, 0.98] | -0.012  [-0.23, -0.0061] | 5.8  [3.0, 114] | N/A | N/A | -0.00011  [-0.014, -8.6e-7] | 6301  [49.5, 8.1e+5] |
|  | 2LTR *env*- | 2.73  [2.51, 2.95] | 0.932  [0.86, 0.96] | -0.022  [-0.052, -0.0093] | 31.5  [13.3, 74.5] | N/A | N/A | -0.000032  [not identified] | 2.2e+4  [not identified] |
| Non-controllers | Intact | 4.28  [3.90, 4.66] | 0.68  [0.46, 0.85] | -0.00075  [-0.0087, -0.000065] | 924  [79.7, 10663] | -0.061  [-0.27, -0.014] | 11.4  [2.6, 49.5] | -0.0038  [-0.0051, -0.0027] | 182  [136, 257] |
|  | HM | 2.57  [2.22, 2.92] | 0.99  [0.1, 0.99] | -0.00043  [-0.068, -2.6e-6] | 1612  [10.2, 2.7e+5] | -0.0048  [-0.0099, -0.0024] | 144  [70.0, 289] | -0.00032  [-5.16, +9.6e-09] | 2166  [0.1, +7.2e+7] |
|  | 2LTR  Total | 3.61  [3.33, 3.89] | 0.87  [not identified] | -0.00034  [-0.0015, -0.00008] | 2039  [462, 8664] | -0.014  [-0.073, -0.0026] | 49.5  [9.5, 267] | -0.00032  [-0.89, -1e-7] | 2166  [0.8, 6.9e+6] |
|  | 2LTR *env*+ | 3.58  [3.25, 3.91] | 0.91  [0.77, 0.97] | -0.00034  [-0.0016, -0.000094] | 2039  [433, 7374] | -0.015  [not identified] | 46.2  [not identified] | -0.00011  [-0.014, -8.6e-7] | 6301  [49.5, 8.1e+5] |
|  | 2LTR *env*- | 2.73  [2.51, 2.95] | 0.87  [0.67, 0.96] | -0.00024  [-3.33, -1.2e-08] | 2888  [0.2, 5.9e+7] | -0.015  [not identified] | 46.2  [not identified] | -0.000032  [not identified] | 2.2e+4  [not identified] |

95% confidence intervals are shown below calculated values in parentheses. Ctrl=control, non-ctrl=non-control. Values with a “+” represent an increase/doubling-time. VL=viral load. HM=hypermutated proviruses. $Y$=variable of interest. $Y_{0}$=baseline value. $A$=fraction of $Y$ that decays in the first-phase post-peak for controllers. $B$=the fraction of $Y$ that decays in the first-phase following ART-initiation for non-controllers. $a_{1}$=first-phase decay rate for controllers. $a_{3}$= first-phase, pre-ART decay rate for non-controllers. $b_{1}$=first-phase decay rate under ART. $a_{2}$=second-phase decay rate. $b_{2}$=second-phase decay rate under ART. See Mathematical Modeling of Decay section of methods for details.
