# Supplementary material for: Dynamics of natural and pharmacologic control of an SIV variant with an envelope trafficking defect
Source: J Exp Med. 2025 Dec 5;223(2):e20251172. doi: 10.1084/jem.20251172 (PMC12679998; doi:10.1084/jem.20251172)
Supplement: Table S2 — shows decay parameters for individual animals from peripheral blood and LNs. [file jem_20251172_tables2.docx]

**Table S2: Decay Parameters for Individual Animals from Peripheral Blood and Lymph Nodes**

| Decay Parameters from Peripheral Blood/Plasma | | | | | | | | | |
| --- | --- | --- | --- | --- | --- | --- | --- | --- | --- |
| $Y$ | Animal ID | Y_0_  (log_10_) | *A* (ctrl) or *B* (non-ctrl) | $a_{1}$ or $a_{3}$ (/day) | 1^st^ phase *t_1/2_* (ctrl) or pre-ART *t_1/2_* (non-ctrl)  (days) | $b_{1}$(/day) | 1^st^ phase post-ART *t_1/2_*  (days) | $a_{2}$ (ctrl) or  $b_{2}$ (non-ctrl) (/day) | 2^nd^ phase *t_1/2_* or *T_d_* (days) |
| Viral Load | NV12 | 5.98  [5.57, 6.38] | 0.999998 [0.99999, 0.99999] | -0.191  [-0.220,  -0.162] | 3.6  [3.2, 4.3] | N/A | N/A | +0.0065 [+0.0005, +0.0125] | +106.6 [+554.5, +55.5] |
|  | NV13 | 6.24  [5.83, 6.66] | 0.999981 [0.99991, 0.99999] | -0.187  [-0.216,  -0.157] | 3.7  [3.2, 4.4] | N/A | N/A | -0.0049  [-0.0107, +0.0010] | 145.5  [64.8, +693.1] |
|  | NV15 | 6.36  [5.95, 6.77] | 0.999952 [0.99965, 0.99999] | -0.154  [-0.184,  -0.123] | 4.5  [3.8, 5.6] | N/A | N/A | -0.0128  [-0.0223,  -0.0033] | 54.2  [31.1, 210.1] |
|  | NV16 | 6.15  [5.76, 6.54] | 0.999986 [0.99992, 0.99999] | -0.146  [-0.175,  -0.117] | 4.8  [4.0, 5.9] | N/A | N/A | -0.0045  [-0.0116, +0.0025] | 154.0  [59.7, +277.3] |
|  | NV20 | 5.72  [5.32, 6.12] | 0.999950 [0.99964, 0.99999] | -0.167  [-0.198,  -0.137] | 4.2  [3.5, 5.1] | N/A | N/A | -0.0079  [-0.0166, +0.0008] | 87.7  [41.7, +866.4] |
|  | NV21 | 5.34  [4.95, 5.74] | 0.999889 [0.99950, 0.99998] | -0.193  [-0.227,  -0.160] | 3.6  [3.1, 4.3] | N/A | N/A | -0.0067  [-0.0138, +0.0005] | 103.4  [50.1, +1386.3] |
|  | NV10 | 6.61  [6.21, 7.01] | 0.99968 [0.99937, 0.99983] | -0.042  [-0.056,  -0.028] | 16.5  [12.4, 24.8] | -0.661  [-0.771,  -0.550] | 1.0  [0.9, 1.3] | -0.0140  [-0.0253,  -0.0027] | 49.5  [27.3, 256.7] |
|  | NV11 | 6.17  [5.75, 6.59] | 0.99972 [0.99945, 0.99985] | -0.052  [-0.067,  -0.036] | 13.3  [10.3, 19.3] | -0.679  [-0.788,  -0.571] | 1.0  [0.9, 1.2] | -0.0107  [-0.0242, +0.0028] | 64.8  [28.9, +247.6] |
|  | NV17 | 6.63  [6.18, 7.08] | 0.99968 [0.99939, 0.99983] | -0.027  [-0.043,  -0.011] | 25.7  [16.1, 63.0] | -0.680  [-0.791,  -0.570] | 1.0  [0.9, 1.2] | -0.0174  [-0.0270,  -0.0079] | 39.8  [25.7, 87.7] |
| Intact Proviruses | NV12 | 4.30  [4.19, 4.41] | 0.84  [0.80,  0.87] | -0.034  [ -0.036,  -0.033] | 20.4  [19.3,  21.0] | N/A | N/A | -0.0072  [-0.0082,  -0.0062] | 96.3  [84.6,  112.7] |
|  | NV13 | 4.58  [4.46, 4.69] | 0.84  [0.80,  0.87] | -0.034  [ -0.036,  -0.032] | 20.4  [19.3,  21.7] | N/A | N/A | -0.0078  [-0.0089,  -0.0068] | 88.9  [77.9,  101.9] |
|  | NV15 | 4.39  [4.28, 4.50] | 0.83  [0.79,  0.86] | -0.034  [ -0.036,  -0.032] | 20.4  [19.3,  21.7] | N/A | N/A | -0.0062  [-0.0072,  -0.0051] | 112.2  [96.2,  134.6] |
|  | NV16 | 4.55  [4.44, 4.66] | 0.83  [0.79,  0.86] | -0.034  [ -0.036,  -0.032] | 20.4  [19.3,  21.7] | N/A | N/A | -0.0063  [-0.0073,  -0.0053] | 109.4  [94.4,  130.0] |
|  | NV20 | 3.68  [3.57, 3.79] | 0.83  [0.79,  0.87] | -0.034  [ -0.036,  -0.033] | 20.4  [19.3,  21.0] | N/A | N/A | -0.0055  [-0.0065,  -0.0045] | 126.2  [106.7,  154.2] |
|  | NV21 | 3.86  [3.74, 3.97] | 0.84  [0.80,  0.88] | -0.034  [ -0.036,  -0.033] | 20.4  [19.3,  21.0] | N/A | N/A | -0.0067  [-0.0078,  -0.0057] | 103.0  [89.3,  121.6] |
|  | NV10 | 4.71  [4.61, 4.80] | 0.32  [0.30,  0.33] | -0.026  [ -0.028,  -0.024] | 26.7  [24.8,  28.9] | -0.0571  [-0.0736,  -0.0406] | 12.1  [9.4,  17.1] | -0.0059  [-0.0070,  -0.0048] | 117.5  [99.2,  143.9] |
|  | NV11 | 4.53  [4.43, 4.62] | 0.32  [0.30,  0.33] | -0.025  [ -0.027,  -0.024] | 27.7  [25.7,  28.9] | -0.0564  [-0.0732,  -0.0395] | 12.3  [9.5,  17.6] | -0.0068  [-0.0079,  -0.0057] | 101.9  [87.6,  122.0] |
|  | NV17 | 5.04  [4.93, 5.14] | 0.32  [0.30,  0.33] | -0.026  [ -0.027,  -0.024] | 26.7  [24.8,  28.9] | -0.0579  [-0.0741,  -0.0417] | 12.0  [9.4,  16.6] | -0.0067  [-0.0078,  -0.0055] | 103.9  [89.0,  125.3] |
| Hypermutated | NV12 | 2.59 [2.45, 2.74] | 0.74  [0.66,  0.80] | -0.033  [-0.035,  -0.031] | 21.0  [19.8, 22.4] | N/A | N/A | -0.00033  [-0.00134, +0.00067] | 2100.5 [517.3, +1033.3] |
|  | NV13 | 2.80 [2.65, 2.95] | 0.76  [0.68,  0.82] | -0.033  [-0.035,  -0.031] | 21.0  [19.8, 22.4] | N/A | N/A | -0.00082  [-0.00183, +0.00018] | 845.3  [378.6, +3850.0] |
|  | NV15 | 2.74 [2.59, 2.89] | 0.77  [0.69,  0.84] | -0.033  [-0.035,  -0.031] | 21.0  [19.8, 22.4] | N/A | N/A | -0.00076  [-0.00179, +0.00026] | 912.0  [387.3, +2661.5] |
|  | NV16 | 2.79 [2.64, 2.94] | 0.75  [0.67,  0.81] | -0.033  [-0.035,  -0.031] | 21.0  [19.8, 22.4] | N/A | N/A | -0.00123  [-0.00225,  -0.00021] | 563.5  [308.3, +3301.5] |
|  | NV20 | 2.47 [2.32, 2.62] | 0.75  [0.67,  0.81] | -0.033  [-0.035,  -0.031] | 21.0  [19.8, 22.4] | N/A | N/A | -0.00074  [-0.00178, +0.00030] | 936.7  [387.3, +2307.7] |
|  | NV21 | 2.48 [2.34, 2.63] | 0.75  [0.67,  0.81] | -0.033  [-0.036,  -0.031] | 21.0  [19.3, 22.4] | N/A | N/A | -0.00008  [-0.00107, +0.00092] | 8664.3 [646.6, +753.0] |
|  | NV10 | 2.84 [2.72, 2.97] | 1.00  [1.00,  1.00] | -0.015  [-0.017,  -0.013] | 46.2  [40.8, 53.3] | -0.00190  [-0.00211,  -0.00170] | 364.7 [327.0, 407.8] | -0.00063  [-0.00187, +0.00060] | 1100.2 [370.6, +1155.0] |
|  | NV11 | 2.52 [2.40, 2.65] | 1.00  [1.00,  1.00] | -0.015  [-0.017,  -0.013] | 46.2  [40.8, 53.3] | -0.00192  [-0.00213,  -0.00171] | 361.1 [322.1, 405.6] | -0.00063  [-0.00186, +0.00059] | 1100.2 [371.6, +1162.7] |
|  | NV17 | 3.26 [3.14, 3.39] | 1.00  [1.00,  1.00] | -0.015  [-0.017,  -0.013] | 46.2  [40.8, 53.3] | -0.00191  [-0.00212,  -0.00170] | 363.0 [325.0, 407.8] | -0.00063  [-0.00187, +0.00060] | 1100.2 [370.6, +1155.0] |
| 2LTR Total | NV12 | 3.68 [3.54, 3.82] | 0.83  [0.78,  0.87] | -0.0260  [-0.0321,  -0.0198] | 26.7  [21.6, 35.0] | N/A | N/A | -0.00444  [-0.00578,  -0.00310] | 156.1  [120.1, 223.5] |
|  | NV13 | 3.89 [3.74, 4.04] | 0.84  [0.79,  0.88] | -0.0249  [-0.0306,  -0.0193] | 27.8  [22.7, 35.9] | N/A | N/A | -0.00576  [-0.00714,  -0.00439] | 120.4  [97.1, 157.8] |
|  | NV15 | 3.70 [3.56, 3.84] | 0.84  [0.79,  0.88] | -0.0250  [-0.0304,  -0.0195] | 27.7  [22.8, 35.5] | N/A | N/A | -0.00416  [-0.00549,  -0.00283] | 166.6  [126.2, 244.7] |
|  | NV16 | 3.95 [3.81, 4.09] | 0.83  [0.78,  0.87] | -0.0248  [-0.0308,  -0.0189] | 27.9  [22.5, 36.7] | N/A | N/A | -0.00489  [-0.00623,  -0.00354] | 141.7  [111.2, 195.8] |
|  | NV20 | 3.01 [2.87, 3.15] | 0.82  [0.77,  0.86] | -0.0270  [-0.0330,  -0.0210] | 25.7  [21.0, 33.0] | N/A | N/A | -0.00301  [-0.00440,  -0.00161] | 230.2  [157.5, 430.8] |
|  | NV21 | 3.30 [3.16, 3.43] | 0.84  [0.79,  0.88] | -0.0271  [-0.0327,  -0.0215] | 25.6  [21.2, 32.2] | N/A | N/A | -0.00373  [-0.00507,  -0.00240] | 185.9  [136.7, 288.9] |
|  | NV10 | 3.81 [3.65, 3.97] | 0.61  [0.48,  0.73] | -0.0138  [-0.0184,  -0.0092] | 50.2  [37.7, 75.3] | -5.00  [-5.60, -4.40] | 0.1  [0.1, 0.2] | -0.00371  [-0.00521,  -0.00220] | 187.0  [133.2, 315.0] |
|  | NV11 | 3.58 [3.41, 3.75] | 0.51  [0.37,  0.64] | -0.0094  [-0.0141,  -0.0047] | 73.7  [49.1, 147.5] | -5.00  [-5.68, -4.32] | 0.1  [0.1, 0.2] | -0.00583  [-0.00732,  -0.00434] | 118.9  [94.6, 159.6] |
|  | NV17 | 4.26 [4.08, 4.44] | 0.57  [0.44,  0.70] | -0.0102  [-0.0153,  -0.0050] | 67.9  [45.3, 138.6] | -5.00  [-5.67, -4.32] | 0.1  [0.1, 0.2] | -0.00650  [-0.00803,  -0.00496] | 107.0  [86.3, 139.8] |
| 2LTR *env*+ | NV12 | 3.63 [3.49, 3.77] | 0.80  [0.77,  0.83] | -0.0281  [-0.0298,  -0.0264] | 24.7  [23.3, 26.3] | N/A | N/A | -0.0050  [-0.0062,  -0.0037] | 138.6  [111.8, 187.5] |
|  | NV13 | 3.82 [3.68, 3.96] | 0.81  [0.77,  0.83] | -0.0280  [-0.0297,  -0.0263] | 24.8  [23.3, 26.4] | N/A | N/A | -0.0065  [-0.0078,  -0.0052] | 106.6  [88.5, 133.3] |
|  | NV15 | 3.60 [3.47, 3.74] | 0.80  [0.77,  0.83] | -0.0281  [-0.0297,  -0.0264] | 24.7  [23.3, 26.3] | N/A | N/A | -0.0046  [-0.0058,  -0.0034] | 150.6  [119.5, 203.8] |
|  | NV16 | 3.89 [3.75, 4.02] | 0.80  [0.77,  0.83] | -0.0280  [-0.0298,  -0.0262] | 24.8  [23.2, 26.5] | N/A | N/A | -0.0054  [-0.0067,  -0.0041] | 128.4  [103.4, 169.1] |
|  | NV20 | 2.96 [2.82, 3.10] | 0.80  [0.77,  0.83] | -0.0281  [-0.0300,  -0.0263] | 24.7  [23.1, 26.4] | N/A | N/A | -0.0041  [-0.0055,  -0.0028] | 169.0  [125.9, 247.7] |
|  | NV21 | 3.24 [3.10, 3.38] | 0.80  [0.77,  0.83] | -0.0281  [-0.0299,  -0.0264] | 24.7  [23.2, 26.3] | N/A | N/A | -0.0055  [-0.0068,  -0.0041] | 126.0  [101.9, 169.1] |
|  | NV10 | 3.67 [3.53, 3.81] | 0.68  [0.53,  0.80] | -0.0101  [-0.0118,  -0.0084] | 68.6  [58.7, 82.5] | -1.08  [-1.64, -0.51] | 0.6  [0.4, 1.4] | -0.0044  [-0.0060,  -0.0028] | 157.5  [115.5, 247.7] |
|  | NV11 | 3.56 [3.41, 3.70] | 0.52  [0.39,  0.66] | -0.0097  [-0.0115,  -0.0078] | 71.4  [60.1, 88.8] | -1.02  [-1.72, -0.33] | 0.7  [0.4, 2.1] | -0.0063  [-0.0079,  -0.0048] | 110.0  [87.8, 144.5] |
|  | NV17 | 4.20 [4.05, 4.35] | 0.61  [0.45,  0.75] | -0.0097  [-0.0115,  -0.0079] | 71.4  [60.1, 87.8] | -1.03  [-1.68, -0.37] | 0.7  [0.4, 1.9] | -0.0064  [-0.0079,  -0.0049] | 108.3  [87.8, 141.2] |
| 2LTR *env*- | NV12 | 2.71 [2.56, 2.86] | 0.86  [0.84,  0.87] | -0.0202  [-0.0226,  -0.0178] | 34.3  [30.7, 38.9] | N/A | N/A | -0.0024  [-0.0028,  -0.0020] | 288.7  [247.5, 346.6] |
|  | NV13 | 2.85 [2.69, 3.01] | 0.86  [0.84,  0.87] | -0.0201  [-0.0225,  -0.0176] | 34.5  [30.8, 39.3] | N/A | N/A | -0.0024  [-0.0028,  -0.0020] | 288.7  [247.5, 346.6] |
|  | NV15 | 2.75 [2.60, 2.91] | 0.86  [0.84,  0.87] | -0.0203  [-0.0227,  -0.0179] | 34.1  [30.5, 38.7] | N/A | N/A | -0.0024  [-0.0028,  -0.0020] | 288.7  [247.5, 346.6] |
|  | NV16 | 2.99 [2.84, 3.14] | 0.86  [0.84,  0.87] | -0.0200  [-0.0225,  -0.0175] | 34.7  [30.8, 39.6] | N/A | N/A | -0.0024  [-0.0028,  -0.0020] | 288.7  [247.5, 346.6] |
|  | NV20 | 2.35 [2.18, 2.52] | 0.86  [0.84,  0.87] | -0.0202  [-0.0226,  -0.0178] | 34.3  [30.7, 38.9] | N/A | N/A | -0.0024  [-0.0028,  -0.0020] | 288.7  [247.5, 346.6] |
|  | NV21 | 2.53 [2.37, 2.70] | 0.86  [0.84,  0.87] | -0.0205  [-0.0229,  -0.0181] | 33.8  [30.3, 38.3] | N/A | N/A | -0.0024  [-0.0028,  -0.0020] | 288.7  [247.5, 346.6] |
|  | NV10 | 2.85 [2.68, 3.01] | 0.69  [0.62,  0.76] | -0.0058  [-0.0081,  -0.0034] | 119.5 [85.7, 204.0] | -1.73  [-3.11, -0.35] | 0.4  [0.2, 2.0] | -0.0024  [-0.0028,  -0.0020] | 288.7  [247.5, 346.6] |
|  | NV11 | 2.54 [2.37, 2.70] | 0.69  [0.62,  0.75] | -0.0056  [-0.0079,  -0.0033] | 123.8 [87.8, 210.0] | -1.72  [-3.24, -0.19] | 0.4  [0.2, 3.6] | -0.0024  [-0.0028,  -0.0020] | 288.7  [247.5, 346.6] |
|  | NV17 | 3.16 [2.99, 3.33] | 0.70  [0.62,  0.76] | -0.0054  [-0.0077,  -0.0030] | 128.3 [90.0, 231.0] | -1.72  [-3.27, -0.16] | 0.4  [0.2, 4.3] | -0.0024  [-0.0028,  -0.0021] | 288.7  [247.5, 333.4] |

| Decay Parameters from Lymph Nodes | | | | | | | | | |
| --- | --- | --- | --- | --- | --- | --- | --- | --- | --- |
| $Y$ | Animal ID | Y_0_  (log_10_) | *A* (ctrl) or *B* (non-ctrl) | $a_{1}$ or $a_{3}$ (/day) | 1^st^ phase *t_1/2_* (ctrl) or pre-ART *t_1/2_* (non-ctrl)  (days) | $b_{1}$(/day) | 1^st^ phase post-ART *t_1/2_*  (days) | $a_{2}$ (ctrl) or  $b_{2}$ (non-ctrl) (/day) | 2^nd^ phase *t_1/2_* or *T_d_* (days) |
| Intact Proviruses | NV12 | 4.73 [4.54, 4.91] | 0.92  [0.90, 0.94] | -0.0214  [-0.0355,  -0.0072] | 32.4  [19.5, 96.3] | N/A | N/A | -0.0039  [-0.0044,  -0.0034] | 177.6  [157.7, 203.9] |
|  | NV13 | 4.64 [4.47, 4.80] | 0.92  [0.90, 0.93] | -0.0338  [-0.0528,  -0.0147] | 20.5  [13.1, 47.1] | N/A | N/A | -0.0038  [-0.0043,  -0.0033] | 182.3  [162.6, 210.2] |
|  | NV15 | 4.96 [4.79, 5.12] | 0.92  [0.90, 0.93] | -0.0410  [-0.0634,  -0.0185] | 16.9  [10.9, 37.5] | N/A | N/A | -0.0037  [-0.0042,  -0.0032] | 187.3  [167.4, 216.6] |
|  | NV16 | 4.74 [4.57, 4.92] | 0.91  [0.89, 0.93] | -0.0470  [-0.0768,  -0.0173] | 14.8  [9.0, 40.0] | N/A | N/A | -0.0037  [-0.0042,  -0.0032] | 187.3  [167.4, 216.6] |
|  | NV20 | 3.95 [3.78, 4.12] | 0.91  [0.89, 0.93] | -0.0549  [-0.0870,  -0.0228] | 12.6  [8.0, 30.4] | N/A | N/A | -0.0037  [-0.0042,  -0.0033] | 187.3  [167.4, 210.2] |
|  | NV21 | 4.24 [4.07, 4.41] | 0.92  [0.89, 0.93] | -0.0394  [-0.0621,  -0.0167] | 17.6  [11.2, 41.5] | N/A | N/A | -0.0037  [-0.0042,  -0.0033] | 187.3  [167.4, 210.2] |
|  | NV10 | 4.32 [4.17, 4.46] | 0.64  [0.54, 0.74] | -0.00072  [-0.00125,  -0.00018] | 963.2  [554.7, 3850.5] | -0.050  [-0.095,  -0.006] | 13.9  [7.3, 115.5] | -0.0037  [-0.0042,  -0.0032] | 187.3  [167.4, 216.6] |
|  | NV11 | 4.02 [3.86, 4.17] | 0.71  [0.61, 0.80] | -0.00077  [-0.00140,  -0.00015] | 899.5  [494.6, 4620.0] | -0.057  [-0.107,  -0.007] | 12.2  [6.5, 98.4] | -0.0038  [-0.0043,  -0.0033] | 182.3  [162.6, 210.2] |
|  | NV17 | 4.49 [4.34, 4.64] | 0.71  [0.61, 0.80] | -0.00077  [-0.00141,  -0.00014] | 899.5  [491.4, 4942.6] | -0.073  [-0.131,  -0.015] | 9.5  [5.3, 46.2] | -0.0038  [-0.0043,  -0.0033] | 182.3  [162.6, 210.2] |
| Hypermutated | NV12 | 3.93 [3.69, 4.17] | 0.9881 [0.9848, 0.9907] | -0.0888  [-0.1310,  -0.0467] | 7.8  [5.3, 14.8] | N/A | N/A | -0.00052  [-0.00181, +0.00077] | 1331.4 [382.8, +903.4] |
|  | NV13 | 4.01 [3.79, 4.23] | 0.9875 [0.9840, 0.9903] | -0.1616  [-0.2448,  -0.0783] | 4.3  [2.8, 8.8] | N/A | N/A | -0.00028  [-0.00100, +0.00044] | 2471.7 [693.1, +5773.7] |
|  | NV15 | 3.85 [3.60, 4.10] | 0.9877 [0.9842, 0.9904] | -0.1420  [-0.2319,  -0.0520] | 4.9  [3.0, 13.3] | N/A | N/A | -0.00041  [-0.00171, +0.00088] | 1689.3 [405.8, +1714.8] |
|  | NV16 | 3.83 [3.59, 4.08] | 0.9880 [0.9846, 0.9907] | -0.1398  [-0.1985,  -0.0810] | 5.0  [3.5, 8.6] | N/A | N/A | -0.00048  [-0.00182, +0.00086] | 1447.2 [381.8, +804.7] |
|  | NV20 | 3.74 [3.54, 3.95] | 0.9878 [0.9844, 0.9905] | -0.3278  [-0.5732,  -0.0825] | 2.1  [1.2, 8.4] | N/A | N/A | -0.00033  [-0.00117, +0.00051] | 2101.5 [590.6, +1350.6] |
|  | NV21 | 3.67 [3.46, 3.88] | 0.9880 [0.9845, 0.9907] | -0.2623  [-0.4131,  -0.1115] | 2.6  [1.7, 6.2] | N/A | N/A | -0.00022  [-0.00071, +0.00027] | 3146.3 [1019.7, +2570.4] |
|  | NV10 | 2.49 [2.30, 2.68] | 0.99998 [0.98804, 1.00000] | -0.0004  [-0.0010, +0.0002] | 1732.9 [693.1, +3465.7] | -0.0043  [-0.0063,  -0.0022] | 161.2 [109.9, 315.0] | -0.00032  [-0.00200, +0.00132] | 2034.6 [348.6, +5453.0] |
|  | NV11 | 2.13 [1.93, 2.34] | 0.99998 [0.99734, 1.00000] | -0.0004  [-0.0011, +0.0002] | 1732.9 [630.9, +3465.7] | -0.0077  [-0.0106,  -0.0048] | 90.0  [65.3, 144.4] | -0.00032  [-0.00205, +0.00141] | 2034.6 [340.4, +6772.5] |
|  | NV17 | 3.11 [2.92, 3.29] | 0.99998 [0.98728, 1.00000] | -0.0004  [-0.0009, +0.0001] | 1732.9 [770.5, +6931.5 | -0.0040  [-0.0059,  -0.0021] | 173.3 [117.5, 330.0] | -0.00032  [-0.00211, +0.00147] | 2034.6 [328.3, +7225.0] |
| 2LTR Total | NV12 | 4.05 [3.83, 4.27] | 0.94 [0.91, 0.96] | -0.0116  [-0.0142,  -0.0089] | 59.7  [48.8, 77.8] | N/A | N/A | -0.00512  [-0.02765, +0.01741] | 135.4  [25.1, +39.8] |
|  | NV13 | 3.62 [3.42, 3.82] | 0.94  [0.91, 0.96] | -0.0124  [-0.0154,  -0.0093] | 55.9  [45.0, 74.5] | N/A | N/A | -0.00086  [-0.00251, +0.00078] | 807.5  [276.9, +889.7] |
|  | NV15 | 3.71 [3.52, 3.90] | 0.94  [0.91, 0.96] | -0.0118  [-0.0148,  -0.0087] | 58.7  [46.8, 79.9] | N/A | N/A | -0.00042  [-0.00168, +0.00083] | 1649.5 [401.5, +835.1] |
|  | NV16 | 4.01 [3.82, 4.20] | 0.94  [0.91, 0.96] | -0.0129  [-0.0162,  -0.0097] | 53.8  [42.8, 70.2] | N/A | N/A | -0.00036  [-0.00140, +0.00069] | 1926.8 [495.0, +1003.5] |
|  | NV20 | 2.91 [2.72, 3.09] | 0.94  [0.91, 0.96] | -0.0119  [-0.0151,  -0.0088] | 58.6  [45.9, 78.7] | N/A | N/A | -0.00025  [-0.00106, +0.00056] | 2772.6 [654.1, +1237.3] |
|  | NV21 | 3.24 [3.06, 3.43] | 0.93  [0.91, 0.95] | -0.0133  [-0.0168,  -0.0097] | 52.2  [41.3, 71.5] | N/A | N/A | -0.00017  [-0.00075, +0.00040] | 4082.5 [922.3, +1733.6] |
|  | NV10 | 3.69 [3.51, 3.86] | 0.88  [0.83, 0.91] | -0.00034  [-0.00044,  -0.00025] | 2038.2 [1575.7, 2773.5] | -0.0132  [-0.0192,  -0.0072] | 52.5  [36.1, 96.3] | -0.00045  [-0.00249, +0.00160] | 1552.3 [277.5, +4335.6] |
|  | NV11 | 3.37 [3.20, 3.55] | 0.88  [0.83, 0.91] | -0.00034  [-0.00044,  -0.00025] | 2038.2 [1575.7, 2773.5] | -0.0152  [-0.0222,  -0.0083] | 45.5  [31.2, 83.5] | -0.00030  [-0.00145, +0.00084] | 2324.5 [673.8, +826.3] |
|  | NV17 | 3.92 [3.75, 4.10] | 0.88  [0.83, 0.91] | -0.00034  [-0.00043,  -0.00025] | 2038.2 [1605.0, 2773.5] | -0.0137  [-0.0199,  -0.0074] | 50.4  [34.8, 93.7] | -0.00041  [-0.00210, +0.00127] | 1649.5 [309.5, +545.2] |
| 2LTR *env*+ | NV12 | 4.02 [3.80, 4.23] | 0.95  [0.90, 0.97] | -0.01147  [-0.01346,  -0.00948] | 60.4  [51.5, 73.1] | N/A | N/A | -0.00555  [-0.07839, +0.06729] | 124.8  [8.8, +10.3] |
|  | NV13 | 3.57 [3.38, 3.76] | 0.95  [0.92, 0.98] | -0.01197  [-0.01433,  -0.00961] | 57.9  [48.4, 72.1] | N/A | N/A | -0.00015  [-0.00133, +0.00102] | 4829.0 [520.6, +679.4] |
|  | NV15 | 3.66 [3.48, 3.85] | 0.95  [0.91, 0.97] | -0.01165  [-0.01402,  -0.00929] | 59.5  [49.4, 74.6] | N/A | N/A | -0.00013  [-0.00127, +0.00101] | 5552.5 [787.4, +670.3] |
|  | NV16 | 3.97 [3.78, 4.16] | 0.95  [0.91, 0.97] | -0.01237  [-0.01488,  -0.00986] | 56.0  [46.6, 70.3] | N/A | N/A | -0.00010  [-0.00087, +0.00067] | 7234.7 [802.3, +1035.8] |
|  | NV20 | 2.87 [2.68, 3.06] | 0.94  [0.89, 0.97] | -0.01180  [-0.01429,  -0.00931] | 58.7  [48.5, 74.6] | N/A | N/A | -0.00009  [-0.00067, +0.00050] | 8040.2 [1492.5, +1380.0] |
|  | NV21 | 3.17 [2.98, 3.36] | 0.94  [0.89, 0.96] | -0.01239  [-0.01500,  -0.00978] | 55.9  [46.2, 70.9] | N/A | N/A | -0.00007  [-0.00056, +0.00041] | 10347.4 [1779.7, 1463.4] |
|  | NV10 | 3.65 [3.48, 3.82] | 0.91  [0.88, 0.93] | -0.00034  [-0.00042,  -0.00027] | 2038.2 [1650.5, 2575.2] | -0.0138  [-0.0192,  -0.0083] | 50.2  [36.1, 83.5] | -0.00010  [-0.00729, +0.00709] | 7234.7  [95.0, +97.2] |
|  | NV11 | 3.36 [3.19, 3.53] | 0.91  [0.88, 0.93] | -0.00034  [-0.00042,  -0.00027] | 2038.2 [1650.5, 2575.2] | -0.0155  [-0.0216,  -0.0095] | 44.7  [32.1, 72.9] | -0.00009  [-0.00082, +0.00064] | 8040.2 [1218.3, +1073.6] |
|  | NV17 | 3.90 [3.73, 4.07] | 0.91  [0.88, 0.93] | -0.00034  [-0.00042,  -0.00027] | 2038.2 [1650.5, 2575.2] | -0.0142  [-0.0196,  -0.0087] | 48.8  [35.4, 79.5] | -0.00011  [-0.00132, +0.00109] | 6578.3 [503.6, +631.9] |
| 2LTR env- | NV12 | 2.93 [2.68, 3.17] | 0.929  [0.914, 0.942] | -0.01392  [-0.02476,  -0.00308] | 49.8  [28.0, 225.0] | N/A | N/A | -0.000032  [-0.000363, +0.000299] | 21659.6 [1909.3, +23108.0] |
|  | NV13 | 2.80 [2.54, 3.06] | 0.929  [0.914, 0.941] | -0.02071  [-0.04067,  -0.00076] | 33.5  [17.0, 913.2] | N/A | N/A | -0.000031  [-0.000292, +0.000229] | 22311.8 [2364.4, +19829.7] |
|  | NV15 | 2.75 [2.47, 3.02] | 0.929  [0.914, 0.941] | -0.01217  [-0.02869, +0.00435] | 57.0  [24.1, +159.3] | N/A | N/A | -0.000031  [-0.000361, +0.000298] | 22311.8 [1909.3, +23218.0] |
|  | NV16 | 2.93 [2.70, 3.16] | 0.930  [0.916, 0.942] | -0.01749  [-0.03070,  -0.00427] | 39.6  [22.6, 162.3] | N/A | N/A | -0.000033  [-0.000418, +0.000352] | 20997.9 [1651.2, +19680.7] |
|  | NV20 | 2.44 [2.18, 2.70] | 0.929  [0.915, 0.941] | -0.04672  [-0.09065,  -0.00279] | 14.8  [7.6, 248.6] | N/A | N/A | -0.000033  [-0.000420, +0.000355] | 20997.9 [1631.0, +19394.4] |
|  | NV21 | 2.56 [2.34, 2.78] | 0.929  [0.914, 0.941] | -0.03005  [-0.05856,  -0.00154] | 23.1  [11.8, 449.4] | N/A | N/A | -0.000030  [-0.000219, +0.000160] | 23070.5 [10531.5, +16237.6] |
|  | NV10 | 2.82 [2.62, 3.02] | 0.872  [0.817, 0.913] | -0.00024  [-0.00059, +0.00010] | 2888.8 [1174.6, +6931.5] | -0.0145  [-0.0189,  -0.0102] | 47.9  [36.7, 67.9] | -0.000032  [-0.000369, +0.000305] | 21659.6 [1778.6, +20143.9] |
|  | NV11 | 2.43 [2.23, 2.63] | 0.867  [0.808, 0.909] | -0.00025  [-0.00061, +0.00012] | 2773.5 [1136.7, +5776.2] | -0.0152  [-0.0198,  -0.0106] | 45.6  [35.0, 65.4] | -0.000031  [-0.000304, +0.000242] | 22311.8 [2214.5, +18616.4] |
|  | NV17 | 2.92 [2.72, 3.12] | 0.867  [0.807, 0.911] | -0.00024  [-0.00060, +0.00012] | 2888.8 [1155.0, +5776.2] | -0.0147  [-0.0191,  -0.0102] | 48.1  [36.3, 67.9] | -0.000031  [-0.000345, +0.000283] | 22311.8 [2113.7, +19855.3] |

95% confidence intervals are shown below calculated values in parentheses. Ctrl=control, non-ctrl=non-control. Values with a “+” represent an increase/doubling-time. VL=viral load. HM=hypermutated proviruses. $Y$=variable of interest. $Y_{0}$=baseline value. $A$=fraction of $Y$ that decays in the first-phase post-peak for controllers. $B$=the fraction of $Y$ that decays in the first-phase following ART-initiation for non-controllers. $a_{1}$=first-phase decay rate for controllers. $a_{3}$= first-phase, pre-ART decay rate for non-controllers. $b_{1}$=first-phase decay rate under ART. $a_{2}$=second-phase decay rate. $b_{2}$=second-phase decay rate under ART. See Mathematical Modeling of Decay section of methods for details.
