# Supplementary material for: Dynamics of natural and pharmacologic control of an SIV variant with an envelope trafficking defect
Source: J Exp Med. 2025 Dec 5;223(2):e20251172. doi: 10.1084/jem.20251172 (PMC12679998; doi:10.1084/jem.20251172)
Supplement: Table S3 — shows primer/probe details. [file jem_20251172_tables3.docx]

**Table S3: Primer/Probe Details**

| Full-Length Sequencing | | | | | |
| --- | --- | --- | --- | --- | --- |
| Amplicon | Primer/Probe Name | Sequence | Fluorophore | Quencher | Reference |
| SMILe A Outer | SMILe A OF | AACAGGGACTTGAAGGAGAGTG | N/A | N/A | This manuscript |
|  | SMILe A OR | GTATATCATTCACTGTCCAGGTC | N/A | N/A | This manuscript |
| SMILe A Inner | SMILe A IF | TTGAAGGAGAGTGAGAGACTCC | N/A | N/A | This manuscript |
|  | SMILe A IR | AGGTCTCTCTTTGTGGCAACTC | N/A | N/A | This manuscript |
| SMILe B Outer | SMILe B OF | GAAGTCCAATTAGGAATACCACAC | N/A | N/A | This manuscript |
|  | SMILe B OR | TTCCTGTAGTGGGAAGATTACTC | N/A | N/A | This manuscript |
| SMILe B Inner | SMILe B IF | TTAGGAATACCACACCCTGCAG | N/A | N/A | This manuscript |
|  | SMILe B IR | GTAGTGGGAAGATTACTCTGCTG | N/A | N/A | This manuscript |
| SMILe C Outer | SMILe C OF | AACATGATCACTACAGAACAAGAG | N/A | N/A | This manuscript |
|  | SMILe C OR | CTCCTCTGCAATTTGTCCAC | N/A | N/A | This manuscript |
| SMILe C Inner | SMILe C IF | GATCACTACAGAACAAGAGATAC | N/A | N/A | This manuscript |
|  | SMILe C IR | CTGCAATTTGTCCACATGAAGG | N/A | N/A | This manuscript |
| SMILe D Outer | SMILe D OF | GTAGAAGACCAGGAAATAAGACAG | N/A | N/A | This manuscript |
|  | SMILe D OR | TTCCTGCTTCGGTTTCCCAAAG | N/A | N/A | This manuscript |
| SMILe D Inner | SMILe D IF | CAGTCACCATTATGTCTGGATTG | N/A | N/A | This manuscript |
|  | SMILe D IR | CAAAGCAGAAAGGGTCCTAACAG | N/A | N/A | This manuscript |
| Sanger sequencing of the above amplicons—Inner PCR primers and: | A Seq F | TGGAGCAGAAGTAGTGCCAG | N/A | N/A | This manuscript |
|  | A Seq R | TAAGGCGACTTTTACAGGCTC | N/A | N/A | This manuscript |
|  | B Seq F | TTACCAAGAAGGCAAGCCATTAG | N/A | N/A | This manuscript |
|  | B Seq R | GTGTGTAGATGTGTAATAGGCC | N/A | N/A | This manuscript |
|  | C Seq F | TAAGCGATGTCAGATCCCAG | N/A | N/A | This manuscript |
|  | C Seq R | TTTGCTCTTGTTCCAAGCCTG | N/A | N/A | This manuscript |
|  | D Seq F | GCTCAGTCCCGAACTTTATTG | N/A | N/A | This manuscript |
|  | D Seq R | TCTGGTATGATGCCTTCTTCC | N/A | N/A | This manuscript |

| Droplet Digital PCR | | | | | |
| --- | --- | --- | --- | --- | --- |
| Amplicon | Primer/Probe Name | Sequence | Fluorophore | Quencher | Reference |
| RM/PTM  RPP30-1 | RPP30-1F | AGGATGCTCCGGGAGTATGTA | N/A | N/A | Bender et al., 2019 |
|  | RPP30-1R | CCTGCTTGTCACCTATATAACAT | N/A | N/A | Bender et al., 2019 |
|  | RPP30-1 probe | TCAAGCTGGGAGACGGAAGAGTCAGT | FAM | ZEN/IABkFQ | Bender et al., 2019 |
| RM/PTM  RPP30-2 | RPP30-2F | ACAGACTCACACAATTTAGG | N/A | N/A | Bender et al., 2019 |
|  | RPP30-2R | ACATTCATGCCACTGCACTC | N/A | N/A | Bender et al., 2019 |
|  | RPP30-2 probe | ACAGGGTCTCACTTTGTTGTCCA | HEX | ZEN/IABkFQ | Bender et al., 2019 |
| SIVmac251/239  IPDA Pol | PolF | GCAGGGATAGAGCACACCTTTG | N/A | N/A | Bender et al., 2019 |
|  | PolR | CTATGGTTTCTACTGAATTTGCTTGTTC | N/A | N/A | Bender et al., 2019 |
|  | Pol intact probe | TTTCAGGTGGTGATTCA | FAM | MGBNFQ | Bender et al., 2019 |
|  | Pol HM probe | TAGGTGGTGATTTATT | N/A | MGBNFQ | Bender et al., 2019 |
| SIVmac239 IPDA Env | EnvF | CCTCAATAAAGCCTTGTGTAAAATTATC | N/A | N/A | Bender et al., 2019 |
|  | 239EnvR | GTTGTTATTGATTTTGTCAATCCC | N/A | N/A | This manuscript |
|  | Env intact probe | TGCATTACTATGAGATGC | VIC | MGBNFQ | Bender et al., 2019 |
|  | Env HM probe | TGCATTACTATAAAATGC | N/A | MGBNFQ | Bender et al., 2019 |
| SIVmac251/239  HPDA Pol | PolF | GCAGGGATAGAGCACACCTTTG | N/A | N/A | Bender et al., 2019 |
|  | PolR | CTATGGTTTCTACTGAATTTGCTTGTTC | N/A | N/A | Bender et al., 2019 |
|  | Pol intact probe | TTTCAGGTGGTGATTCA | N/A | MGBNFQ | Fray et al., 2023 |
|  | Pol HM probe | TAGGTGGTGATTTATT | FAM | MGBNFQ | Fray et al., 2023 |
| SIVmac239 HPDA Env | EnvF | CCTCAATAAAGCCTTGTGTAAAATTATC | N/A | N/A | Bender et al., 2019 |
|  | 239EnvR | GTTGTTATTGATTTTGTCAATCCC | N/A | N/A | This manuscript |
|  | Env intact probe | TGCATTACTATGAGATGC | N/A | MGBNFQ | Fray et al., 2023 |
|  | Env HM probe | TGCATTACTATAAAATGC | VIC | MGBNFQ | Fray et al., 2023 |
| 2LTR Circles | 2-LTRc F | CGCCTGGTCAACTCGGTACTC | N/A | N/A | Policicchio et al., 2018 |
|  | 2-LTRc R | GGTATGATGCCTTCTTCCTTTTCTAAG | N/A | N/A | Policicchio et al., 2018 |
|  | 2-LTRc probe | CCCTGGTCTGTTAGGACCCTTTCTGCTTTG | FAM | MGBNFQ | Policicchio et al., 2018 quencher modified in Bender et al., 2019 |

| Barcode & Envelope Single Genome Sequencing | | | | | |
| --- | --- | --- | --- | --- | --- |
| Amplicon | Primer/Probe Name | Sequence | Fluorophore | Quencher | Reference |
| Outer | BarcodeEnv OF | ATAAACAGAGAGGCGGTAAACCACC | N/A | N/A | This manuscript |
|  | SIVsm/macEnvR1 | TGTAATAAATCCCTTCCAGTCCCCCC | N/A | N/A | Keele et al., 2009 |
| Inner | BarcodeEnv IF | GTAAACCACCTACCAAGGGAGCTAAT | N/A | N/A | This manuscript |
|  | SIVsmEnvR2 | ATGAGACATRTCTATTGCCAATTTGTA | N/A | N/A | Keele et al., 2009 |
| Sanger sequencing of the above amplicons—Inner PCR primers and: | SMILe C IR | CTGCAATTTGTCCACATGAAGG | N/A | N/A | This manuscript |
|  | SMILe D IF | CAGTCACCATTATGTCTGGATTG | N/A | N/A | This manuscript |
|  | C Seq R | TTTGCTCTTGTTCCAAGCCTG | N/A | N/A | This manuscript |
|  | D Seq F | GCTCAGTCCCGAACTTTATTG | N/A | N/A | This manuscript |
|  | Xtra Env 5 | TAATGAGTTCTCCTGCTCCCTC | N/A | N/A | This manuscript |
|  | Xtra Env 3 | ATCTGCGACAGAGACTCTTGC | N/A | N/A | This manuscript |
| cDNA Synthesis & Plasma Virus Sequencing | | | | | |
| Amplicon | Primer/Probe Name | Sequence | Fluorophore | Quencher | Reference |
| cDNA synthesis | SIVsm/macEnvR1 | TGTAATAAATCCCTTCCAGTCCCCCC | N/A | N/A | Keele et al., 2009 |
| Full Env Outer | BarcodeEnv OF | ATAAACAGAGAGGCGGTAAACCACC | N/A | N/A | This manuscript |
|  | SIVsm/macEnvR1 | TGTAATAAATCCCTTCCAGTCCCCCC | N/A | N/A | Keele et al., 2009 |
| Full Env Inner | BarcodeEnv IF | GTAAACCACCTACCAAGGGAGCTAAT | N/A | N/A | This manuscript |
|  | SIVsmEnvR2 | ATGAGACATRTCTATTGCCAATTTGTA | N/A | N/A | Keele et al., 2009 |
| Sequencing | Same as for proviral SGS | | | | |
